# Data-driven detection of subtype-specific differentially expressed genes

**DOI:** 10.1038/s41598-020-79704-1

**Published:** 2021-01-11

**Authors:** Lulu Chen, Yingzhou Lu, Chiung-Ting Wu, Robert Clarke, Guoqiang Yu, Jennifer E. Van Eyk, David M. Herrington, Yue Wang

**Affiliations:** 1 Department of Electrical and Computer Engineering, Virginia Polytechnic Institute and State University, Arlington, VA 22203 USA; 2 Lombardi Comprehensive Cancer Center, Georgetown University, Washington, DC 20057 USA; 3 Advanced Clinical Biosystems Research Institute, Cedars Sinai Medical Center, Los Angeles, CA 90048 USA; 4 Department of Internal Medicine, Wake Forest University, Winston-Salem, NC 27157 USA

**Keywords:** Computational models, Machine learning, Software, Statistical methods

## Abstract

Among multiple subtypes of tissue or cell, subtype-specific differentially-expressed genes (SDEGs) are defined as being most-upregulated in only one subtype but not in any other. Detecting SDEGs plays a critical role in the molecular characterization and deconvolution of multicellular complex tissues. Classic differential analysis assumes a null hypothesis whose test statistic is not subtype-specific, thus can produce a high false positive rate and/or lower detection power. Here we first introduce a One-Versus-Everyone Fold Change (OVE-FC) test for detecting SDEGs. We then propose a scaled test statistic (OVE-sFC) for assessing the statistical significance of SDEGs that applies a mixture null distribution model and a tailored permutation test. The OVE-FC/sFC test was validated on both type 1 error rate and detection power using extensive simulation data sets generated from real gene expression profiles of purified subtype samples. The OVE-FC/sFC test was then applied to two benchmark gene expression data sets of purified subtype samples and detected many known or previously unknown SDEGs. Subsequent supervised deconvolution results on synthesized bulk expression data, obtained using the SDEGs detected from the independent purified expression data by the OVE-FC/sFC test, showed superior performance in deconvolution accuracy when compared with popular peer methods.

## Introduction

Molecular characterization often applies gene expression profiling to a complex biologic system that includes some molecular features that are expressed by all cell or tissue types in the system (such as housekeeping genes)^1^ and other features that are specific to one or more cell or tissue subtypes present in the system (marker genes or differentially-expressed genes)^2–4^. An important but frequently underappreciated issue is how best to define a cell or tissue subtype-specific expression pattern. Ideally, a subtype-specific expression pattern would be composed of individual features that are most-upregulated in the cell or tissue subtype of interest while in no others (subtype-specific differentially expressed genes, SDEGs)^5–8^.

SDEGs play a critical role in molecularly characterizing and identifying tissue or cell subtypes. For example, to support supervised deconvolution of complex tissues^5,8,9^, the expression patterns of detected SDEGs could serve as the supervising information. However, detecting SDEGs using molecular expression profiles of purified/isolated tissue or cell subtypes remains a challenging task^10^. For example, the most frequently used methods rely on the extension of an ANOVA model where the null hypothesis states that samples in all subtypes are drawn from the same population. Consequently, ANOVA detects genes differentially expressed across any of the subtypes and can identify many false positive SDEGs (subtype-nonspecific classic DEGs) that may not conform to the SDEG definition (Supplementary Information). One-Versus-Rest Fold Change (OVR-FC) is another popular method based on the ratio of the average expression in a particular subtype to that of the average expression in all other samples (rest)^10–12^, and OVR t-test is occasionally used to assess the statistical significance of the detected genes^13^. However, a gene with a low average expression value in the rest is not necessarily expressed at a low level in every subtype in the rest. Expectedly, simulation studies show that Marker Gene Finder in Microarray data (MGFM) outperforms OVR t-test^14^. Alternative strategies include One-Versus-One (OVO) t-test and Multiple Comparisons with the Best (MCB)^15^ that use additional pairwise significance testing or the confidence intervals of OVO statistics^2,16^.

To address the critical problem of the absence of a detection method explicitly matched to the definition of SDEGs, here we introduce One-Versus-Everyone Fold Change (OVE-FC) test to detect SDEGs among many subtypes. Previously, the OVE-FC test was proposed as a means to detect SDEGs and improve multiclass classification, where the selection is based on whether the mean of one subtype is significantly higher or lower than the mean from each of the other subtypes^5,6^. To assess the statistical significance of such a test, we propose a scaled test statistic (OVE-sFC) together with a mixture null distribution model. Because the expression patterns of non-SDEGs can be highly complex, a tailored permutation test is used to estimate the corresponding distribution under the null hypothesis.

**Figure 1:**
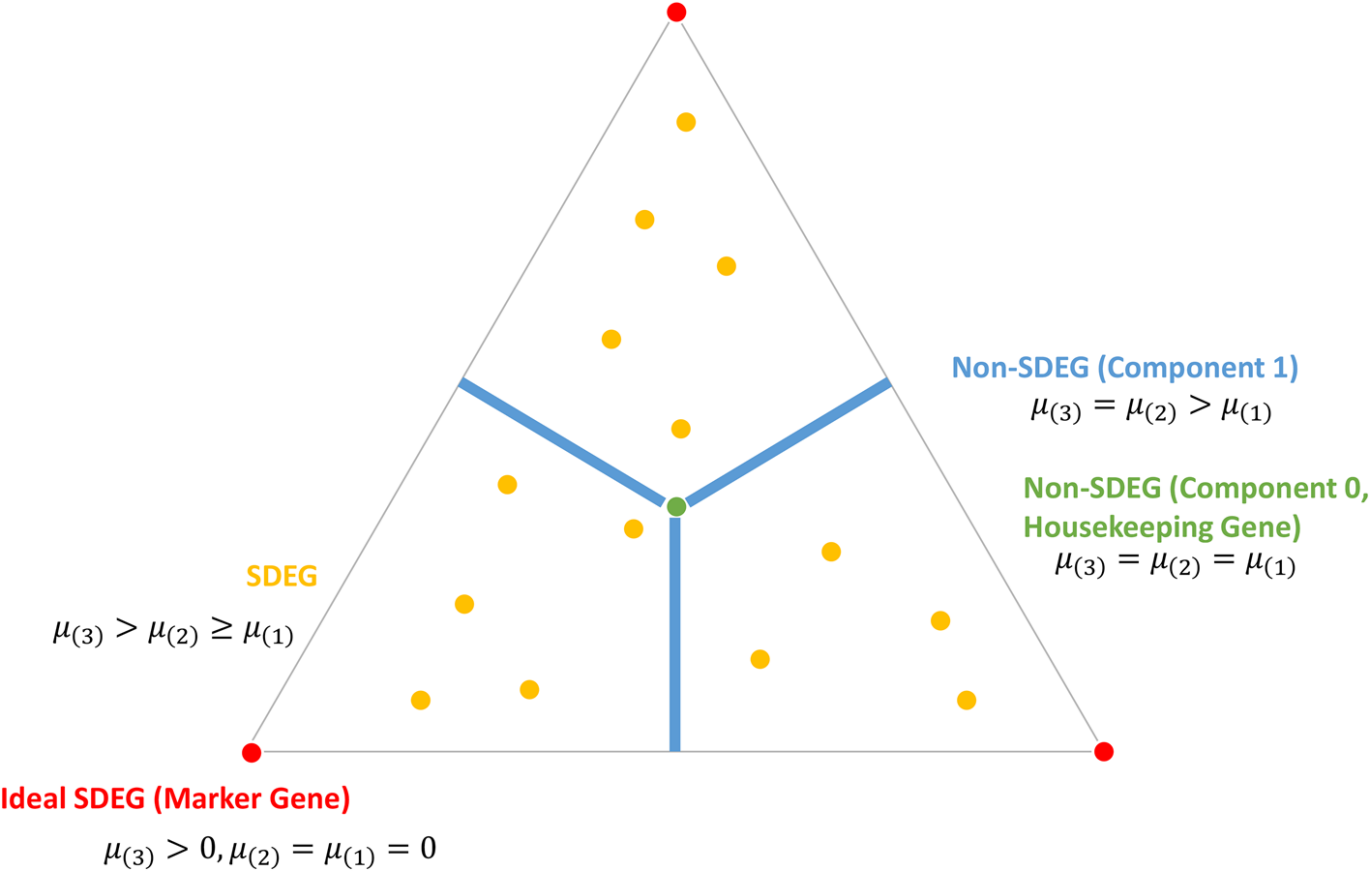
Illustrative simplex of three subtypes. Given the definition of SDEGs, and for simplicity, consider a scenario where three subtypes have the mean values ${\mu }_{(1)}\le {\mu }_{\left(2\right)}\le {\mu }_{(3)}$ and define ${d}_{j}={\mu }_{\left(3\right)}-{\mu }_{(2)}$. The SDEGs and non-SDEGs can be illustrated in a simplex plot, where yellow/red points are SDEGs under the alternative hypothesis ${d}_{j}>0$ and blue/green points are non-SDEGs under null hypothesis ${\mathrm{d}}_{\mathrm{j}}=0$. OVE-sFC is to test whether ${\mathrm{d}}_{\mathrm{j}}$ is significantly larger than zero and thus matches the definition of SDEG.

Consider the measured expression level ${s}_{k}(i,j)$ of gene $j$ in sample $i$ across $k=1,\dots ,\dots K$ subtypes. We denote the mean and variance of the logarithmic expression levels $\mathrm{log}{s}_{k}\left(i,j\right)$ of gene $j$ in subtype $k$ by ${\mu }_{k}\left(j\right)$ and ${\sigma }^{2}(j)$, respectively. OVE-FC after the logarithm for gene $j$ is defined as the difference between the log2-transformed expression value in the two subtypes where *j* is expressed at the highest and second highest levels, respectively^5,14^,

1 $$\begin{array}{c}{d}_{j}=\underset{l\ne \left(K\right)}{\mathrm{min}}\left\{{\mu }_{\left(K\right)}(j)-{\mu }_{l}(j)\right\},\end{array}$$

and where subscript $(K)$ indicates the subtype with the maximum mean among all subtypes. Note that OVE-FC has previously been proposed for multiclass classification^13,14^, and matches well the definition of SDEGs^5,8,17,18^. Conceptually, the null hypothesis for non-SDEGs, and the alternative hypothesis for SDEGs, can be described as (see Fig. 1)

2 $$\begin{array}{c}\begin{array}{c}{H}_{\text{SDEG}}^{null}{:}\,{ d}_{j}=0;\\ {H}_{\text{SDEG}}^{\text{alt}}{:}\,{ d}_{j}>0.\end{array}\end{array}$$

SDEG corresponds to the above null hypothesis that ${d}_{j}$ = 0, because every expression pattern of non-SDEGs satisfies ${d}_{j}$ = 0. Please find more detailed explanations with a toy example in Supplementary Information. Ideal SDEGs detected by the OVE strategy with a stringent threshold are also used as the marker genes for supervised deconvolution^8,17^, and are similar to what is detected by the Convex Analysis of Mixtures (CAM) method for fully unsupervised deconvolution^9,19^ (marker genes that reside near the vertices of the scatter simplex). To assess the statistical significance of OVE-FC tests and to leverage the information across subtypes or genes, we assume that $\mathrm{log}{s}_{k}\left(i,j\right)\sim N\left({\mu }_{k}\left(j\right),{\sigma }^{2}(j)\right)$ and further define the scaled test statistic OVE-sFC as

3 $$\begin{array}{c}{t}_{j}=\underset{l\ne \left(K\right)}{\mathrm{min}}\left\{\frac{{\mu }_{\left(K\right)}\left(j\right)-{\mu }_{l}\left(j\right)}{\sigma \left(j\right)\sqrt{\frac{1}{{n}_{\left(K\right)}}+\frac{1}{{n}_{l}}}}\right\},\end{array}$$

where ${n}_{\left(K\right)}$ and ${n}_{l}$ are the numbers of samples in subtypes $\left(K\right)$ and $l$, respectively. Modeling the distribution of ${t}_{j}$ under the null hypothesis is challenging for more than two subtypes $K\ge 3$ because the expression patterns of non-SDEGs are highly complex; non-SDEGs include both housekeeping genes and various combinatorial patterns of differentially-expressed genes among the subtypes (see “Methods” section).

We first validate the performance of OVE-sFC test on extensive simulation data, in terms of type 1 error rate and False Discovery Rate (FDR) control. We then demonstrate the detection power of OVE-FC/sFC in a comprehensive set of scenarios and in comparison with top peer methods using the partial area under the receiver operating characteristic curve (pAUC) as the performance measure. We show the utility of OVE-FC/sFC using benchmark public data, and then assess performance both by comparing with known SDEGs and by the accuracy of supervised deconvolution that uses the expression patterns of de novo SDEGs detected by OVE-FC/sFC.

This work aims solely to detect SDEGs among multiple subtypes of interest, no bulk experiment or dataset is involved. All tests use the molecular expression data derived from purified/isolated subtypes, where the subtype can be a tissue subtype, cell subtype, or biological process. The supervised deconvolution case study provides one application that uses SDEGs detected as explicitly defined. The main objective of the proposed OVE test is to reduce the high false positive rates of existing methods while ensuring high sensitivity, particularly when involving a large number of multiple tests.

## Results

### Validation of OVE-sFC test on type 1 error using simulation data sets

To test whether our OVE-sFC test can detect SDEGs at appropriate significance levels, we assessed the type 1 error using simulation studies under the null hypothesis (“Methods” section). Accuracy of type 1 error is crucial for any hypothesis testing methods that detect SDEGs based on their *p* values. If the type 1 error is either too conservative or too liberal, the *p* value is inflated by either too many false positive or false negative estimates, the test loses its intended meaning, and the data become difficult to interpret correctly.

**Figure 2:**
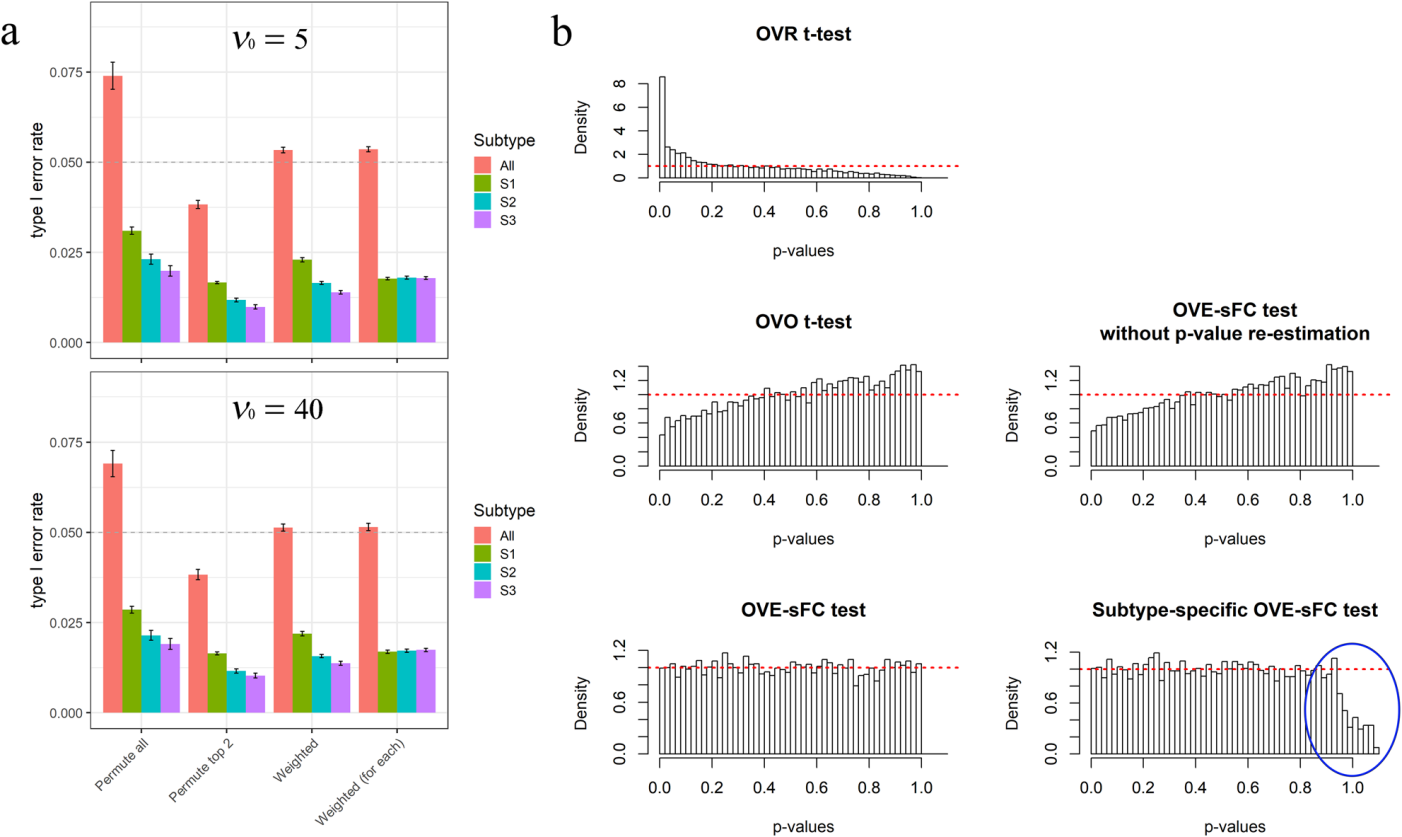
Assessment on Type 1 error rates and *p* value distributions using simulated data sets under the null hypothesis, involving three subtypes with unbalanced sample sizes . 10,000 non-SDEGs are simulated with a portion of housekeeping genes taking the baseline expression levels across all the three subtypes. The remaining non-SDEGs are adjusted to exhibiting similar upregulations in two subtypes. The sample size per subtype is 3, 6, and 9. (**a**) Bar chart for the mean and 95% confidence interval of type I error rates with *p* value cutoff at 0.05 over 150 simulation-based experiments, showing both overall and subtype-specific false-positive rates corresponding to different permutation schemes. 30 parameter settings, with 5 replicates for each, adopted varying housekeeping gene percentages (95%, 80%, 60%, 40%, or 25%), different prior degrees of freedom ${\nu }_{0}$ (5 or 40), and ${\sigma }_{0}$ values (0.2, 0.5, or 0.8). (**b**) Histograms of *p* value distributions associated with the five SDEG detection methods, where simulation data consisted of 60% housekeeping genes, ${\sigma }_{0}=0.5$ and ${\nu }_{0}=40$. Note that subtype-specific *p* values can be higher than 1.0 after multiple testing correction and thus will be truncated (indicated by the blue circle; see Supplementary Information for details).

**Figure 3:**
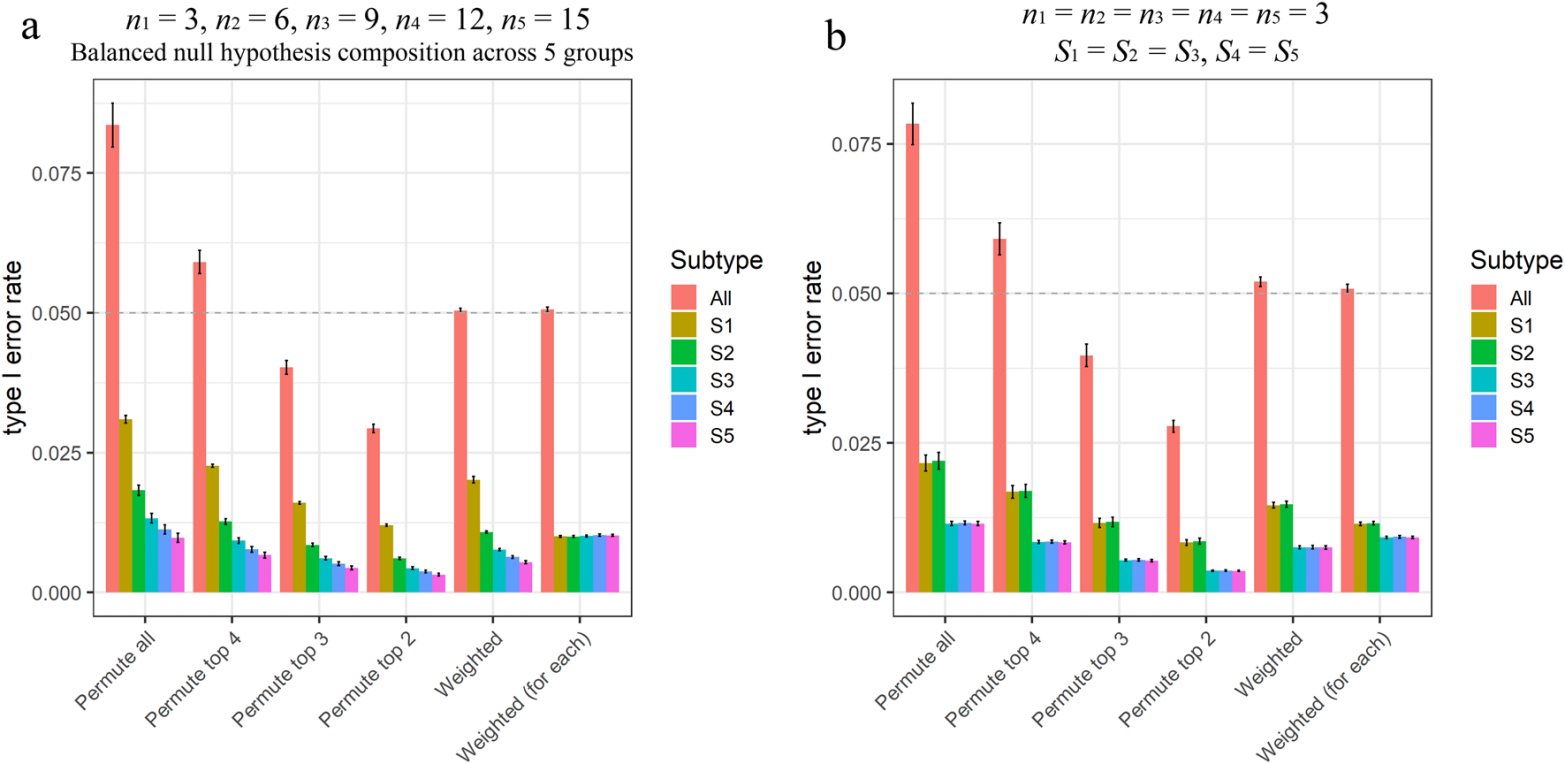
Assessment on Type 1 error rates using simulation data sets involving five subtypes. The results are obtained using the *p* value cutoff at 0.05 over 150 simulation experiments. 10,000 non-SDEGs are simulated with 30 parameter settings and 5 replicates for each. (**a**) Bar chart of the mean and 95% confidence interval of type I error rates with unbalanced sample sizes. A portion of housekeeping genes take the baseline expression levels across all the five subtypes. The remaining non-SDEGs are adjusted to exhibiting similar upregulations in at least two subtypes. The sample size for subtype S1–S5 is n_1_ = 3, n_2_ = 6, n_3_ = 9, n_4_ = 12 and n_5_ = 15, respectively. (**b**) Bar chart of the mean and 95% confidence interval of type I error rates with unbalanced compositions of mixture null distribution. Five subtype-specifc profiles are derived from the real gene expression data of two cell lines,where two subtypes are associated with one cell line and other three subtypes are associated with another cell line, making two subtypes closer to each other and other three close to each other. The data are under the null hypothesis thus no SDEGs exist in any of the five subtypes. The sample size is 3 for each subtype.

In our study, real gene expression data of purified/isolated subtypes were used to create the simulation data sets. A flexible simulation program was written to generate the simulation data sets according to user-defined parameter settings. The approach used ensures that the simulation data retain the basic patterns of the real gene expression data (“Methods” section, and Supplementary Information). In the simulation study to validate type 1 error (or FDR control), we varied the parameter settings in the experiments to observe the impact of these parameters on the performance of various methods, such as varying the noise level and the percentage of housekeeping genes. To validate the OVE-sFC test on type 1 error, the simulation data contained 10,000 genes where baseline expression levels were sampled from benchmark microarray gene expression data with replicates collected from purified cell subtypes (GSE19380^8^). Using the simulation data sets with various parameter settings, we show that in all scenarios the empirical type 1 error produced by OVE-sFC test closely approximates the expected type 1 error (Figs. 2a, 3a,b, S2). The* p* values associated with OVE-sFC test statistics exhibit the expected uniform distribution. Even with unbalanced sample sizes among the subtypes, the mixture null distribution estimated by our posterior-weighted permutation scheme produces the expected empirical type 1 error rate (Figure S2 and Fig. 3a). In contrast, the empirical type 1 error produced by the OVR t-test and the OVO t-test either over-estimates or under-estimates the expected type 1 error. Moreover, the *p* values associated with the OVR t-test and the OVO t-test deviate from a uniform distribution (Fig. 2b). We also evaluated the type 1 error associated with each individual subtypes under high noise levels and using small sample sizes. For each of these subtypes, experimental results show that the empirical type 1 error produced by OVE-sFC test closely matches the expected type 1 error (Fig. 2b and Supplementary Information).

We conducted similar validation studies using five subtypes over a wide range of simulation scenarios (Fig. 3). Experimental results again show that the OVE-sFC test produces empirical type 1 error rates that match the expected type 1 error rates. Furthermore, subtype-specific *p* value estimates effectively balance the uneven type 1 error rates among the subtypes with different numbers of upregulated genes (“Methods” section, Fig. 3b, and Supplementary Information).

### Comparative assessment of OVE-FC/sFC test on power of detecting SDEGs using simulation data sets

Using real gene expression data sets (both microarray and RNAseq data), we simulated a comprehensive set of scenarios to compare the power of OVE-sFC and peer methods to detect SDEGs. Simulation data are again generated by modifying the expression levels of real gene expression data, where a portion of the genes are designated as SDEGs that are upregulated specifically in one of the participating subtypes, with fold change drawn in certain ranges. To recapitulate the characteristics of real expression data, we used parameter values that are close to that estimated from real data, such as proportions of various non-SDEG expression patterns. To retain the mean–variance trend in RNAseq data, we sampled variance directly from the real RNAseq data (“Methods” section and Supplementary Information).

False Discovery Rate (FDR) control is an important issue when assessing detection power in large-scale multiple testing. For a well-designed significance test, the objective is to maximize power while maintaining the FDR below an acceptable level. To test whether the q-value reflects the actual FDR, ‘fdrtool’ was used to estimate the q-value for each gene^20^. The empirical FDR with an estimated q-value of 0.05 is expected to be around 0.05. Another informative criterion is the pAUC that emphasizes the leftmost partial area under the receiver operating characteristic curve, focusing on the sensitivity at lower False Positive Rates (FPR)^21^.

Experimental results show that both overall and subtype-specific OVE-sFC tests achieve a well-controlled FDR that matches the q-value cutoff (Figure S5, S6). In contrast, OVR t-test underestimates, while OVO t-test overestimates, the FDR (Supplementary Information). Subtype-specific OVE-sFC exhibits a more balanced FPR for SDEGs across subtypes, while peer methods produce higher FPRs in the subtypes of smaller sample sizes.

**Figure 4:**
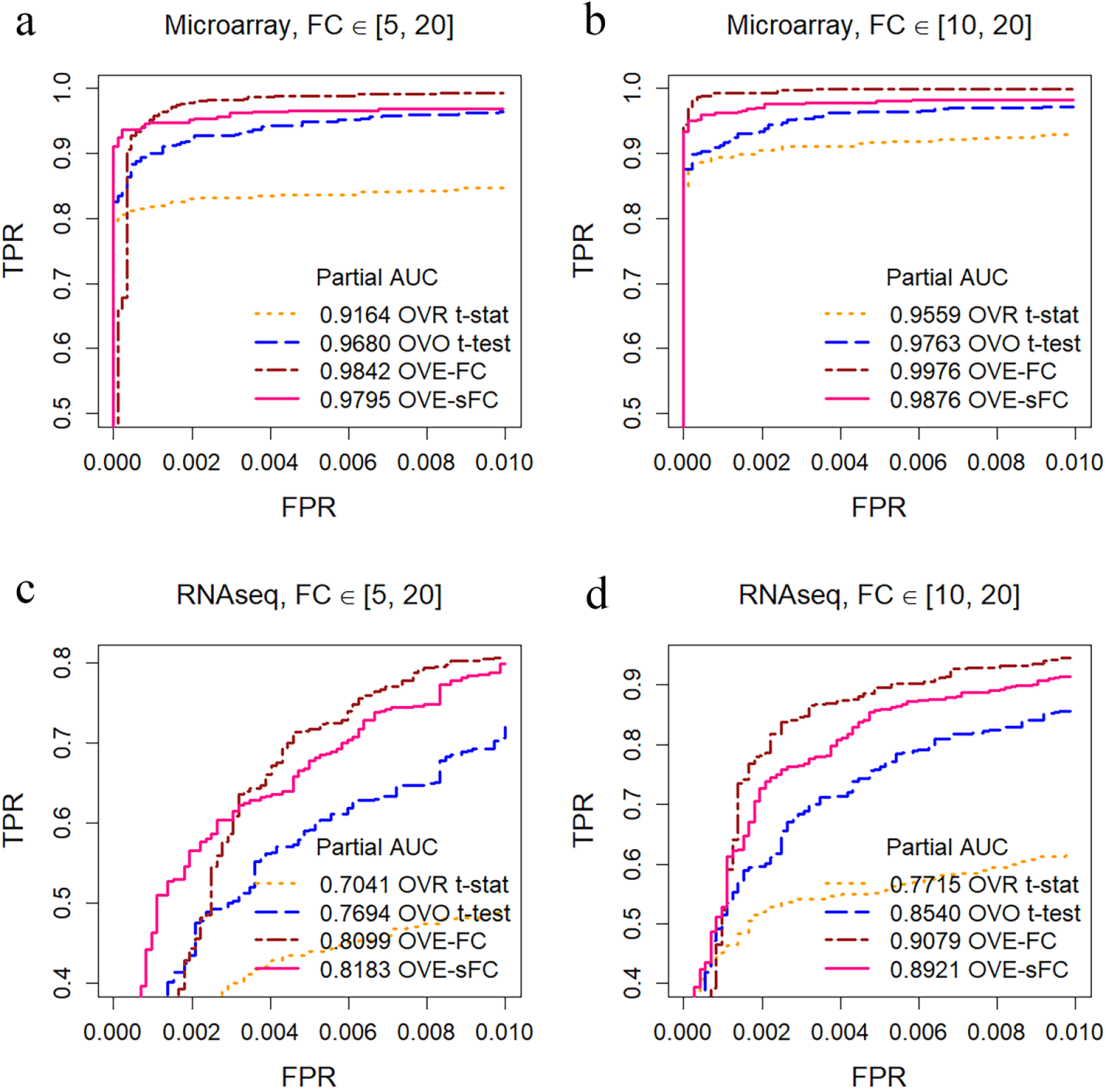
Comparative assessment on detection power (partial ROC curves, FPR < 0.01) using simulations produced from real gene expression data (non-SDEG pattern distribution is consistent with the baseline real dataset under null hypothesis; variances are sampled from real microarray data GSE28490 or RNAseq data GSE60424 with keeping mean–variance trend) involving seven unbalanced subtypes with various parameter settings. SDEGs are adjusted to exhibiting upregulations with varying fold changes sampled from [5, 20] or [10, 20]. (**a**) and (**b**) Partial ROC curves across different FPR points on microarray-derived data. (**c**) and (**d**) partial ROC curves across different FPR points on RNAseq-derived data. (OVR-FC and OVR t-test are not shown here due to low pAUC; subtype-specific OVE-sFC test’s performance is quite similar to OVE-sFC test; more complete ROC curves can be found in Figure S7; more fold change settings can be found in Figure S8). Both OVE-FC and OVE-sFC achieve a better performance than the other methods. OVE-FC achieve the best performance for ideal SDEGs with extremely large fold change, and OVE-sFC would be the preferred choice for SDEGs with small fold change or low SNR (RNASeq data is noiser than microarray data).

For pAUC, the OVE strategy in OVE-FC/sFC achieved the highest power in detecting SDEGs (Figs. 4, S7, S8, Table S1–S3), as demonstrated by our simulations with different fold change ranges and two different data types (microarray—low noise level; RNASeq—high noise level). Specifically, for detecting less-stringent SDEGs (with a sufficiently large fold change, Fig. 4a,c), OVE-sFC would be the preferred choice. For ideal SDEGs (marker genes that exhibit significantly large fold change^8,17^, Fig. 4b,d), both OVE-FC and OVE-sFC achieve the best performance, with a slightly better performance by OVE-FC. In comparison with peer methods, OVE-sFC consistently outperforms OVO t-test in the more challenging experiments that use RNAseq data. The improved performance of OVE-FC/sFC over the peer methods at a stringent FPR range in ROC analysis is important because the related FDR is problematic in many real-world applications where large scale multiple comparisons are involved. In contrast, all three OVR methods exhibit lower detection power; ANOVA has the lowest detection power.

While the OVE test aims to reduce high false positive rates produced by the existing methods, it shall also ensure high detection power (sensitivity). While the percentage of ideal SDEGs is expected to be small when compared with that of non-SDEGs, a high TPR is required to ensure sufficient accuracy of a supervised deconvolution and subtype enrichment analysis (two major utilities of SDEGs). The experimental results shown that, with the desired > 0.9 sensitivity as seen in Fig. 4a,b, peer methods produced much higher false positive rates than OVE. In Figure S8 (with the lower end of SDEG fold-change starting 2, 3, 4), our experimental results show that OVE-sFC clearly outperforms OVO t-test in all scenarios with different effect size, and OVE-FC clearly outperforms all other peer methods except OVO t-test only when the effect size is very small. Since OVE-FC neither considers the variance term in the test nor borrows the relevant/useful information cross genes in estimating null distribution, OVE-FC expectedly underperforms OVO t-test when the effect and/or sample size is small. We developed OVE-sFC for this reason and also to estimate the significance level for the FDR control (Supplementary Information).

When the sample size is small, the OVE-sFC test statistic leverages information across genes by estimating a priori variance via the limma method. This approach stabilizes the variance estimate for each gene. Furthermore, the OVE-sFC test statistic estimates the parameters of the limma model from all subtypes, producing better results than by applying a t-test independently with the limma model for each subtype pair. For small sample size cases, our results show that OVE-sFC clearly outperforms OVO t-test (Figs. 4, 6c and Tables S2, S3). Note that when a large number of genes is involved, a more stringent multiple comparison correction or FPR/FDR control is applied.

### Application of OVE-sFC test on two benchmark gene expression data sets detects SDEGs (human immune cells)

To detect SDEGs associated with human immune cells, we applied the OVE-sFC test to two gene expression microarray data sets acquired from isolated/purified subtypes, GSE28490 (Roche) and GSE28491 (HUG)^22^. The constituent subtypes are composed of seven different human immune cells that were isolated from healthy human blood: B cells, CD4 + T cells, CD8 + T cells, NK cells, monocytes, neutrophils, and eosinophils. Because Roche and HUG used the same protocols for cell isolation and sample processing from two independent panels of donors, the derived gene expression profiles allow the use of a cross-validation strategy.

**Figure 5:**
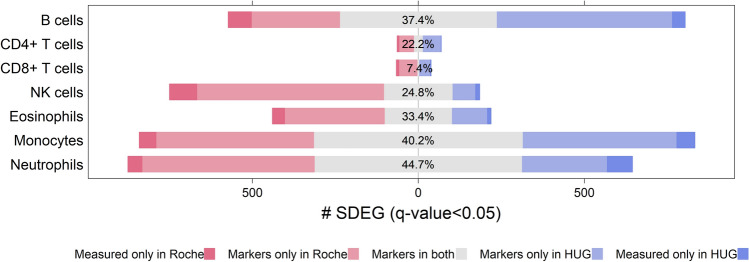
Percentile overlap of cell-type specific SDEGs between Roche and HUG datasets, quantified by Jaccard index (intersection over union). SDEGs are detected by subtype-specific OVE-sFC test with q-value < 0.05.

With an FDR control of q-value < 0.05 applied to both data sets, the OVE-sFC test detects n = 28 CD4 + T cell marker genes, n = 7 CD8 + T cell marker genes, and multiple marker genes for other more distinctive cell types (Tables S4–S6). Between the two data sets, we obtain a Jaccard index (intersection over union) of 36.8% for all SDEGs across all seven cell types. Overlap of monocyte and neutrophil marker genes detected from the two datasets is > 40% (Fig. 5). The number of SDEGs accounts for approximately one-third of all probesets (Roche: 39%, HUG: 34%). This result is expected because these subtypes are pure cell types and so more distinctive than would be seen with samples from complex multicellular tissues^9,19,23^. We also applied a Bonferroni multiple testing correction and a more stringent *p* value < 0.001; the number of SDEGs account for 10.7% and 2.7% of all probesets in the Roche and HUG data sets, respectively (Table S4), with only one common CD4 + T cell marker gene (FHIT) and one common CD8 + T cell marker gene (CD8B).

Figure S9 shows the many combinatorial upregulation patterns among cell types observed under the null hypothesis. Probeset-wise posterior probabilities of component hypotheses in the null mixtures (Eq. 4) were accumulated and normalized to estimate the probabilities of the alternative hypotheses (Eq. S10). The patterns of upregulation in B cells, monocytes, or neutrophils rank the top in both data sets, followed by upregulation in lymphoid cells (B cells, CD4 + T cells, CD8 + T cells, NK cells) and T cells (CD4 + T cells, CD8 + T cells) in the Roche dataset.

### Evaluation of ideal SDEGs detected by OVE-FC/sFC test via supervised deconvolution

Accurate and reliable detection of ideal SDEGs has a significant impact on the performance of many supervised deconvolution methods that use the expression patterns of ideal SDEGs to score constituent subtypes in heterogeneous samples^19,24,25^. We adopted a Convex Analysis of Mixtures (CAM) score calculated from ideal SDEGs-guided supervised deconvolution to quantify the proportional abundance of each subtype (Supplementary Information). The correlation coefficient between the estimated scores and the true proportions was used to assess the accuracy of several SDEGs selection methods.

Both OVE-FC and OVE-sFC were applied to three independent data sets acquired from the purified subtype expression profiles (GSE28490 Roche), purified subtype RNAseq profiles (GSE60424), and classified single-cell RNAseq profiles (GSE72056), respectively. Ideal SDEGs were detected by six different methods including OVE-FC, OVE-sFC, OVR-FC, OVR t-stat, OVR t-test, and OVO t-test, and then used to supervise the deconvolution of realistically synthesized mixtures with ground truth.

**Figure 6:**
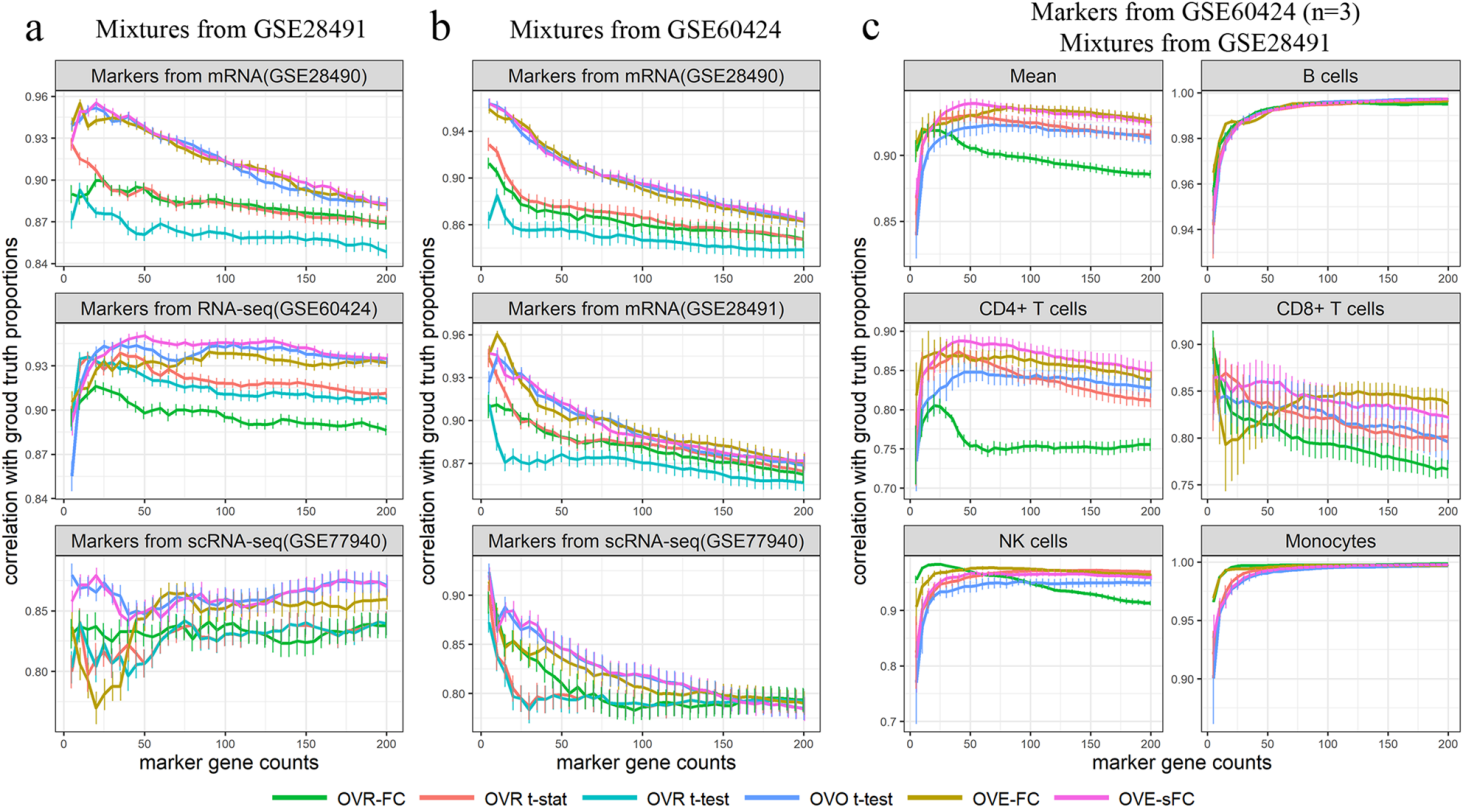
Correlation coefficients between CAM scores and ground truth proportions in simulated heterogeneous samples of mixed subtype mRNA expression profiles or RNAseq counts (**a**–**c** based on three different real gene expression datasets). CAM scores are estimated using the detected SDEGs from independent dataset and reflect the proportions of subtypes (Supplementary Information). The mean and 95% confidence interval are computed over 20 repeated experiments (OVR t-test results are not shown in (**c**) due to very poor performance).

The proportions of constituent subtypes were estimated by the CAM scores derived from expression levels of top-ranked SDEGs for each subtype. Supervised deconvolution results show that OVE-sFC, OVE-FC and OVO t-test achieved the highest correlation coefficients between the CAM score and the true proportions when compared with other methods (Figs. 6a, S10).

To create a more biologically realistic case involving higher between-sample variations, we synthesized a set of n = 50 in silico mixtures by combining the subtype expression profiles from bootstrapped samples in the RNAseq data set according to pre-determined proportions. Again, supervised deconvolution results show that the ideal SDEGs detected by OVE-FC or OVE-sFC or OVO t-test achieved superior deconvolution performance (Figs. 6b, S11).

Using the more challenging case of RNAseq data (lower SNR and small sample size), we repeated the simulations where in silico mixtures were synthesized by combining subtype mean expressions (GSE28491 HUG); ideal SDEGs were detected from the downsampled RNAseq profiles in GSE60424 (n = 3). Three purified samples were randomly selected for each subtype and analyzed by the six methods. In terms of ideal SDEG-guided deconvolution, OVE-sFC strongly outperforms OVO t-test. OVE-sFC also outperforms OVE-FC for phenotypically closer cell types (CD4 + T and CD8 + T cell types) (Fig. 6c).

Across the varying number of ideal SDEGs (5–200) selected, Fig. 6 shows the impact of SDEGs (both at a fixed number and the corresponding content) selected by different methods on the performance of supervised deconvolution. Different subtypes are expected to have different numbers of ideal SDEGs practically and biologically, for example e.g., B cell or monocyte versus CD4 + T cell or CD8 + T cell. The fundamental working principle of many tissue deconvolution methods is that there is a small number of ideal SDEGs expressed unique to each subtype. Thus applying a stringent OVE-sFC test *p* value threshold, such as *p* < 0.001 after correction (Table S4), is a good option because a suitable number of ideal SDEGs for CD4 + or CD8 + T cells is 5–20, while B cells or monocytes often allow a larger number of ideal SDEGs to be used in supervised deconvolution.

## Discussion

Interpreting an expression profile of complex tissues requires knowledge of both the relative abundance of the different cell or tissue subtypes and their unique molecular characteristics. Understanding the relative contribution of individual cell or tissue subtypes in complex samples can illuminate pathophysiologic mechanisms, biologic responses to various stimuli, or transitions in phenotype—especially when cell–cell and cell–matrix interactions in a complex system are necessary conditions for appropriate cell or tissue function. The SDEG expression patterns of relevant cells or tissues can be used to support supervised deconvolution to estimate the relative prevalence of these cell or tissue subtypes. Our present work on SDEGs is restricted to the widely adopted SDEG definition^7,8,17,18^, motivated by the need to obtain such SDEGs to supervise in silico tissue deconvolution^19^ and/or tissue subtype characterization^9^. This is a particulary important goal where the measured data are mixtures of the genes expressed by many of the subtypes present in the samples and the SDEGs are used to estimate both the proportions of each subtype in individual heterogeneous samples and the averaged subtype-specific expression profiles.

While ideal SDEGs are defined as being uniquely and consistently expressed in a tissue or cell subtype across varying conditions, the variability inherent in many tissue samples requires a more relaxed definition that allows the SDEGs of a specific tissue or cell subtype to exhibit low or insignificant expression values in all other subtypes. We show that SDEGs detected by OVE-FC/sFC using high thresholds or small *p* values can accurately estimate both subtype proportions and expression profiles; thus, these SDEGs can serve as effective molecular markers (Figs. 6c, S10 and S11). Accuracy of OVE-FC/sFC-based SDEG detection may be affected by batch effects, normalization, and outliers present in the expression data. Hence, the reliability of OVE-sFC depends on the variance estimate, particularly when sample size is small. In practice, the number of available purified subtype samples are usually small (often 3–5) and is one of the challenges in the problem we are addressing. OVE-sFC integrates the related information across genes or subtypes. For example, OVE-sFC estimates the variances from all subtypes, whereas OVO t-test conducts estimations for only each subtype pair. Consequently, OVE-sFC outperforms OVO t-test, especially in those challenging cases with higher expression variability, smaller sample size, and the presence of a greater number of subtypes (Figs. 4, 6c).

The three major factors affecting the robustness of SDEG detection are noise level (within-subtype expression variability/variance across samples), sample size, and differential expression (fold change) between two subtypes expressed at the highest levels. Our experimental results show that OVE-sFC test maintains type 1 error rates closely matched to the expectations with varied effects (Figure S2), and tends to exhibit better performance than other tools when the noise level is higher and both sample size and fold change are smaller (Fig. 4).

In our study, most of the assumptions applied are widely accepted because they are close to reality. In the simulation study to validate the type 1 error rate produced by OVE-sFC under the null hypothesis, a uniform distribution of the empirical *p* values of OVE-sFC is assumed. This assumption holds when all genes are non-SDEGs and the estimate of the null distribution is sufficiently accurate. OVE-sFC works best when all assumptions in the model are valid. For example, while the proposed permutation scheme does not require the data to be normally distributed under the null hypothesis, OVE-sFC assumes that samples are drawn from a distribution with the same ‘shape’ for different genes. This assumption ensures that the null distributions across genes can be combined with variance-based standardization. When data distributions deviate significantly from a common shape, limma-voom/vooma/voomaByGroup variance models can be used to accommodate unequal variances by appropriate observational-level weights^26^. When data distributions deviate significantly from normality, a permutation ANOVA can be used to estimate the null hypothesis components of the mixture distribution. Figure S6 shows that with the mean–variance relationship estimated by limma-voom on RNASeq data, OVE-sFC can maintain the expected type 1 error rates or specified FDR. For outliers and drop-out zero values in RNAseq data, state-of-the-art two-group test methods designed specifically for RNAseq such as edgeR^27^ and DESeq2^28^ can be adopted when needed.

While OVE-FC is the simpler version of our OVE strategy and drives the OVE-sFC approach described here, we have also demonstrated that OVE-FC is an effective and robust method for detecting SDEGs, particularly when sample size is small. OVE-sFC is a critical complement to OVE-FC. Firstly, OVE-FC does not assess statistical significance (no *p* values are estimated) while OVE-sFC provides a significance assessment and can improve FDR control. Secondly, OVE-sFC improves detection power in some of the more challenging experimental conditions. Detecting SDEGs with accurate *p* values is an attractive feature of OVE-sFC that can help restrain the FDR to its expected level. Indeed, our experimental results show that OVE-sFC test outperforms OVE-FC in the more challenging cases involving nonideal SDEGs (Figure S8) or cell types that are closely related phenotypically (Fig. 6c). However, OVE-sFC test may become unstable when the scaling factor is too small or estimated inaccurately. OVE-FC will not perform well when pre-exclusion of extremely lowly-expressed genes is not done correctly.

ANOVA has been the most commonly used method to test differences among the means of multiple subtypes, often in conjunction with a post-hoc Tukey HSD test to compare all possible pairs of means^29^. However, this approach is not suitable for detecting SDEGs because the null hypothesis used by ANOVA does not truly enforce the definition of SDEGs. ANOVA detects all significant differentially expressed genes rather than the unique subset that represents SDEGs. Hence, an ANOVA model produces too many false positives with respect to individual subtypes (Supplementary Information).

In addition to the SDEGs discussed here (genes uniquely up-regulated in a specific subtype), the counterpart of subtype-specific down-regulated genes (genes uniquely down-regulated in a specific subtype) are also of biological interest^5^. OVE-FC/sFC can detect down-regulated SDEGs by reversing the comparison rule^5^. There are alternative definitions of ‘informative genes’ for different analytical purposes, such as when the goal is sample classification. In our earlier work on multiclass classification^5,6^, we have shown that upregulated SDEGs selected by OVE-FC are sufficient to achieve multiclass classification and can often improve classifier performance over alternative informative gene subsets of the same size.

In the present study, we have chosen to introduce a method focused on univariate analysis. Our method does not consider the network structure among the genes or gene sets. For the future work, we will explore the possibility of networked SDEG detection, laveraging the latest advances in gene set analysis approaches based on multivariate tests^30^.

Lastly, when subtype-specific expression patterns are unknown, unsupervised deconvolution techniques such as CAM^19^ are required. An advantage of unsupervised deconvolution is that it can identify both the cell/tissue subtype proportions and their specific expression patterns, albeit with potentially less fidelity, when neither is known a priori or measured from the same sample.

## Methods

### OVE-sFC test statistic and null distribution modeling

**Figure 7:**
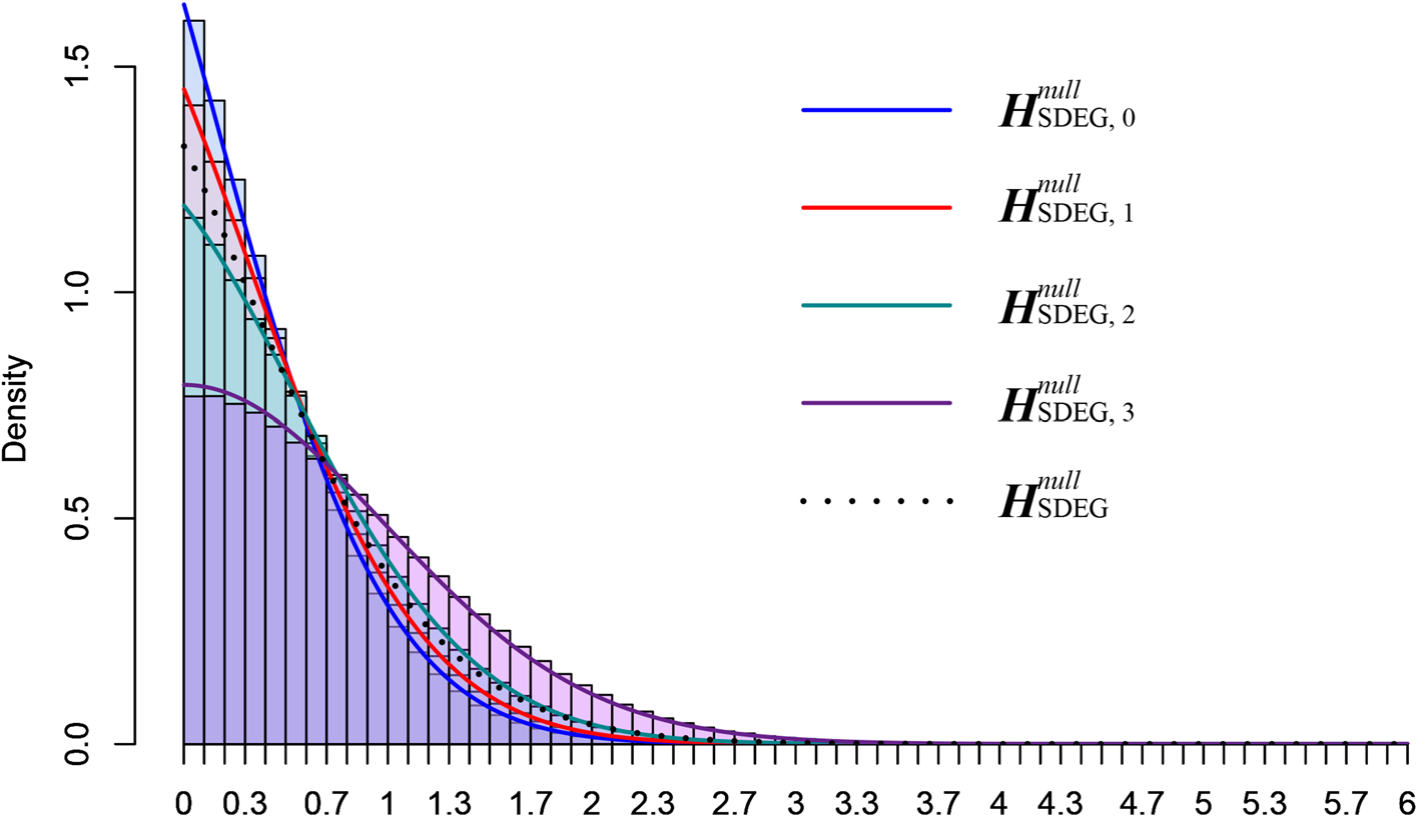
Mixture null distribution of OVE-sFC test statistic for detecting SDEGs. The mixture distribution consists of $(K-1)$ null components, each estimated from the resamples after randomly permuting samples in the top $\left(K-m\right)$ subtypes of high mean expressions and weighted by the posterior probabilities of component null hypotheses.

We propose the following mixture distribution of the OVE-sFC test statistic $t$ under the null hypothesis (Fig. 7)

4 $$\begin{array}{c}f\left\{t|{H}_{\text{SDEG}}^{null}\right\}=\sum_{m=0}^{K-2}f\left\{t|{H}_{\text{SDEG}}^{null,m}\right\}P\left\{{H}_{\text{SDEG}}^{null,m}|{H}_{\text{SDEG}}^{null}\right\},\end{array}$$

where ${H}_{\text{SDEG}}^{null,m}$ is the *m*th component of the mixture null hypothesis ${H}_{\text{SDEG}}^{null}$. We designed a novel nested permutation scheme that approximates the complex null distribution and is consistent with the definition of SDEGs. ${H}_{\text{SDEG}}^{null,m}$ is constructed by permuting the samples in the top $\left(K-m\right)$ subtypes with higher mean expressions; the samples in the bottom $m$ subtypes with lower mean expressions are removed from the permutation. Note that ${H}_{\text{SDEG}}^{null,0}$ corresponds to the same null distribution used in ANOVA where all samples participate in the permutation.

This mixture null distribution model is proposed to model unknown but potentially complex expression patterns of non-SDEGs under the null hypothesis. The permutation scheme(s) estimates such a mixture null distribution. The main advantage of the proposed permutation scheme(s) is its flexibility and comprehensiveness, which closely match the mixture null distribution of various types and combinations. With varying proportions of different non-SDEG types, the OVE-sFC test can maintain the type 1 error rate close to the expected level with the help of the proposed permutation scheme(s) and the conditional probability of each non-SDEG type (Figure S2, Supplementary Information).

Note that ${H}_{\text{SDEG}}^{null,m},m=0,\dots ,K-2$ represents $(K-1)$ different null hypotheses, each with an individualized null distribution that can be estimated by specific permutation scheme(s); essentially, we permute samples in the top $(K-m)$ subtypes. Collectively, a mixture of null distributions is constructed from combinations of different null hypotheses in various proportions. In contrast, without conditioning on ${H}_{\text{SDEG}}^{null,m}$, all null distributions are aggregated equally into the mixture null distribution in the same proportion. This simpler permutation scheme produces an equal-weight mixture model that cannot represent the complexity of the null distribution. Thus, the null distribution of the OVE-sFC test statistic could be distorted. As a result, a uniform distribution of *p* values in null data is not guaranteed and the observed False Discovery Rate may not match the expected level.

The null distribution of OVESEG-test statistics under ${H}_{\text{null-SDEG, }m}$ is estimated from permuted samples and aggregated from different genes with weights. Let ${\varvec{s}}\left(j\right)=[s\left(1,j\right),\dots ,s\left(N,j\right)]$ denote the measured expression vector of gene $j$ across samples, where $N$ is the total number of samples. These weights are the posterior probabilities of a component null hypothesis given the observation $\mathrm{Pr}\left\{{H}_{\text{null-SDEG, }m}|{\varvec{s}}(j)\right\}$, estimated by the local FDR ${\text{fdr}}_{\text{non-SDEG, }m}\left(j\right)$^31^, given by

5a $${w}_{\text{non-SDEG, }0}\left(j\right)=\mathrm{Pr}\left\{{H}_{\text{SDEG}}^{null,0}|{\varvec{s}}\left(j\right)\right\}={\text{fdr}}_{\text{non-SDEG, 0}}\left(j\right),$$

5b $${w}_{\text{non-SDEG, }m}\left(j\right)=\mathrm{Pr}\left\{{H}_{\text{SDEG}}^{null,m}|{\varvec{s}}\left(j\right)\right\}=\left\{1-\sum_{n=0}^{m-1}{w}_{\text{non-SDEG, }n}\left(j\right)\right\}{\text{fdr}}_{\text{non-SDEG, }m}\left(j\right), 0<m<K-2,$$

where ${\text{fdr}}_{\text{non-SDEG, 0}}\left(j\right)$ is the local FDR associated with ANOVA on all subtypes, and ${\text{fdr}}_{\text{non-SDEG, }m}\left(j\right)$ is the local FDR associated with ANOVA on the top $\left(K-m\right)$ subtypes, estimated using R package “fdrtool”^20^ (Supplementary Information).

### Assessing statistical significance of candidate SDEGs

The *p* values of candidate SDEGs are estimated using the learned ‘mixture’ null distribution

6 $$\begin{array}{c}p{\text{-}}value=\mathrm{Pr}\left\{T>{t}_{obs}|{H}_{\text{SDEG}}^{null}\right\}=\sum_{m=0}^{K-2}\mathrm{Pr}\left\{T>{t}_{obs}|{H}_{\text{SDEG}}^{null,m}\right\}P\left\{{H}_{\text{SDEG}}^{null,m}|{H}_{\text{SDEG}}^{null}\right\},\end{array}$$

where ${t}_{obs}$ is the observed OVE-sFC test statistic, and $T$ is the continuous dummy random variable. Specifically, $\mathrm{Pr}\left\{T>{t}_{obs}|{H}_{\text{SDEG}}^{null,m}\right\}$ is calculated by the weighted permutation scores

7 $$\begin{array}{c}\mathrm{Pr}\left\{T>{t}_{obs}|{H}_{\text{SDEG}}^{null,m}\right\}=\frac{{\sum }_{p=1}^{P}{\sum }_{j=1}^{J}{w}_{\text{non-SDEG, }m}\left(j\right)I\left({T}_{j,p}>{t}_{obs}\right) \, }{P{\sum }_{j=1}^{J}{w}_{\text{non-SDEG, }m}\left(j\right) \, },\end{array}$$

where *P* is the number of permutations, *J* is the number of participating genes, $I\left(\cdot \right)$ is the indicator function, and ${T}_{j,p}$ is the OVE-sFC test statistic in the $p$ th permutation on $j$ th gene. Furthermore, the component weight in the mixture null distribution is estimated by the membership expectation of the posterior probabilities over all genes

8 $$\begin{array}{c}P\left\{{H}_{\text{SDEG}}^{null,m}|{H}_{\text{SDEG}}^{null}\right\}=\frac{\sum_{j=1}^{J}{w}_{\text{non-SDEG, }m}\left(j\right)}{\sum_{j=1}^{J}\sum_{n=0}^{K-2}{w}_{\text{non-SDEG, }n}\left(j\right)}.\end{array}$$

Lastly, substituting (7) and (8) into (6), the *p* value associated with gene *j* is calculated by:

9 $$\begin{array}{c}p{\text{-}}value=\frac{\sum_{m=0}^{K-2}{\sum }_{p=1}^{P}{\sum }_{j=1}^{J}{w}_{\text{non-SDEG, }m}\left(j\right)I\left({T}_{j,p}>{t}_{obs}\right) \, }{P\sum_{m=0}^{K-2}\sum_{j=1}^{J}{w}_{\text{non-SDEG, }m}\left(j\right)},\end{array}$$

with a lower bound of ${\mathrm{min}_{j}}\left\{\sum_{m=0}^{K-2}{w}_{\text{non-SDEG, }m}\left(j\right)\right\}/P\sum_{m=0}^{K-2}\sum_{j=1}^{J}{w}_{\text{non-SDEG, }m}\left(j\right)$. Supplementary Information provides more details on the deviation of OVE-sFC test *p* values when considering all subtypes together (Eq. 9) and when considering one subtype specifically (Eq. S7, S8).

### Empirical Bayes moderated variance estimator of within-subtype expressions

The importance of an accurate estimator on pooled within-subtype variance ${\sigma }^{2}(j)$ is twofold—calculating the OVE-sFC test statistic ${t}_{j}$ and determining the local false discovery rate ${\text{fdr}}_{\text{non-SDEG, }m}\left(j\right)$, particularly with a small sample size. We assume a scaled inverse chi-square prior distribution ${\sigma }^{2}(j)\sim {\nu }_{0}{\sigma }_{0}^{2}/{\mathcal{X}}_{{\nu }_{0}}^{2}$, where ${\nu }_{0}$ and ${\sigma }_{0}^{2}$ are the prior degrees of freedom and scaling parameter, respectively^32^. We then adopt the empirical Bayes moderated variance estimator ${\tilde{\sigma }}^{2}(j)$ that leverages information across all genes, as used in *limma* and given by

10 $$\begin{array}{c}{\tilde{\sigma }}^{2}\left(j\right)=\frac{{\nu }_{0}{\widehat{\sigma }}_{0}^{2}+\left(N-K\right){\widehat{\sigma }}^{2}\left(j\right)}{{\nu }_{0}+N-K},\end{array}$$

where *N* is the total number of samples, and ${\widehat{\sigma }}^{2}(j)$ is the pooled variance estimator, given by

11 $$\begin{array}{c}{\widehat{\sigma }}^{2}\left(j\right)=\frac{{\sum }_{k=1}^{K}{\sum }_{i=1}^{{N}_{k}}{\left(\mathrm{log}{s}_{k}\left(i,j\right)-{\mu }_{k}\left(j\right)\right)}^{2}}{N-K}.\end{array}$$

The prior parameters ${\nu }_{0}$ and ${\sigma }_{0}^{2}$ are estimated from the pooled variances. The moderated variances shrink the pooled variances towards the prior values depending on the prior degrees of freedom and the number of samples. Note that $t\text{-}stat\left(j\right)$ with moderated variance estimator ${\tilde{\sigma }}^{2}\left(j\right)$ follows a *t*-distribution with ${\nu }_{0}+N-K$ degrees of freedom (Supplementary Information).

### Brief review of the most relevant peer SDEG selection methods

The OVR-FC uses a simple test defined by

12 $$\begin{array}{c}{\text{OVR-FC}}_{k}\left(j\right)=\frac{{\bar{s}}_{k}\left(j\right)}{{\bar{s}}_{-k}\left(j\right)},\end{array}$$

where ${\bar{s}}_{k}\left(j\right)$ and ${\bar{s}}_{-k}\left(j\right)$ are the geometric means of the $j$th gene expressions within subtype $k$ and associated with the combined remaining subtypes, respectively. The OVR t-test uses a statistical test given by

13 $$\begin{array}{c}{{\rm OVR}}\,{\text{t-stat}}_{k}\left(j\right)=\frac{{\widehat{\mu }}_{k}\left(j\right)-{\widehat{\mu }}_{-k}\left(j\right)}{\sqrt{\frac{{\widehat{\sigma }}_{k}\left(j\right)}{{n}_{k}}+\frac{{\widehat{\sigma }}_{-k}\left(j\right)}{{N-n}_{k}}}},\end{array}$$

where ${\widehat{\mu }}_{k}\left(j\right)$ and ${\widehat{\mu }}_{-k}\left(j\right)$ are the sample means of the $j$th gene expressions within subtype $k$ and associated with the combined remaining subtypes, respectively; ${n}_{k}$ is the number of samples in subtypes $k$; and ${\widehat{\sigma }}_{k}\left(j\right)$ and ${\widehat{\sigma }}_{-k}\left(j\right)$ are the sample variances of the th gene expressions within subtype $k$ and associated with the combined remaining subtypes, respectively. The OVO t-test conducts t-tests among all subtype pairs and selects genes upregulated in one subtype for all the tests, where the variances are estimated only from every pair of subtypes^16^ (Supplementary Information). In contrast, OVE-sFC exploits all subtypes in estimating the variances. The benefit of using all subtypes for modeling is significant in challenging cases with higher variance, smaller sample size, and more subtypes (Supplementary Information).

### Simulation study for validating OVE-sFC test statistics on type 1 error

Among the 10,000 simulated genes, a portion are housekeeping genes that take the baseline expression levels across all subtypes under ${H}_{\text{SDEG}}^{null,0}$. The expression levels of the remaining genes are proportionally adjusted to exhibit similar levels of upregulation as seen in at least two subtypes depending on *m* values, mimicking all types of non-SDEGs under the participating null hypotheses ${H}_{\text{SDEG}}^{null,m>0}$. The mean upregulation levels are drawn from a properly bounded uniform distribution in scatter space, with variance following an inverse chi-square distribution ${\sigma }^{2}(j)\sim {\nu }_{0}{\sigma }_{0}^{2}/{\mathcal{X}}_{{\nu }_{0}}^{2}$, where the prior degree of freedom ${\nu }_{0}$ takes 5 or 40, and ${\sigma }_{0}$ takes 0.2, 0.5, or 0.8 (Supplementary Information).

### Simulation study for asscessing OVE-FC/sFC on the power of detecting SDEGs

Among the 10,000 simulated genes, ratios of non-SDEG patterns were consistent with the estimation from the base real dataset; microarray data GSE28490 or RNAseq data GSE60424. 100 SDEGs were mimicked with their upregulations sampled from a fold change range [5, 20], or [10, 20]. Variances were sampled from base real dataset according to gene expression levels, preserving the potential mean–variance trend (Supplementary Information).

### Gene expression data of human immune cells (GSE28490 and GSE28491)

In these data sets, each cell subtype consists of at least five samples, excluding a few outliers (Table S7). Following preprocessing of the raw measurements, 12,022 probesets in Roche and 11,339 probesets in HUG were retained and used in the analyses (Supplementary Information).

### Realistic synthetic data for supervised deconvolution

Five subtypes (B cell, CD4 + T cell, CD8 + T cell, NK cell, monocytes) were included in synthesizing n = 50 in silico mixtures, where purified subtype mean expression data from the GSE28491 HUG dataset were combined according to pre-determined proportions with additive noise, simulating heterogeneous biological samples (Supplementary Information).

## Supplementary Information

Supplementary Infomations.

## Data Availability

A Bioconductor approved R package of OVE-sFC is freely available at http://bioconductor.org/packages/OVESEG. A detailed user’s manual and a vignette are provided within the package. In addition, public gene expression data analyzed in this paper are also available from the Gene Expression Omnibus Database under Accession Number GEO: GSE19380, GSE28490, GSE28491, GSE60424, and GSE72056.
