# Supplementary material for: Data-driven detection of subtype-specific differentially expressed genes
Source: Sci Rep. 2021 Jan 11;11:332. doi: 10.1038/s41598-020-79704-1 (PMC7801594; doi:10.1038/s41598-020-79704-1)
Supplement: Supplementary file 1 — Supplementary Infomations. [file 41598_2020_79704_MOESM1_ESM.docx]

**Supplementary Information**

Data-driven detection of subtype-specific differentially expressed genes

Lulu Chen^1^, Yingzhou Lu^1^, Chiung-Ting Wu^1^, Robert Clarke^2^, Guoqiang Yu^1^, Jennifer E. Van Eyk^3^, David M. Herrington^4,*^, and Yue Wang^1,*^

^1^Department of Electrical and Computer Engineering, Virginia Polytechnic Institute and State University, Arlington, VA 22203, USA; ^2^Lombardi Comprehensive Cancer Center, Georgetown University, Washington, DC 20057, USA; ^3^Advanced Clinical Biosystems Research Institute, Cedars Sinai Medical Center, Los Angeles, CA 90048, USA; ^4^Department of Internal Medicine, Wake Forest University, Winston-Salem, NC 27157, USA

^*^Author for correspondence: Yue Wang, Ph.D.

Virginia Tech Research Center - Arlington

900 N. Glebe Road, Arlington, VA 22203

E-mail: yuewang@vt.edu

## Toy example to understand SDEG definition and its null hypothesis

We first use a toy example to illustrate the expression patterns of SDEGs according to the given definition. For simplicity of discussion, we will focus the illustration on the SDEGs most-upregulated in only one subtype while in no others, and use expression patterns of ideal SDEGs (red points in **Fig. 1**) to represent all possible expression patterns of SDEGs (red and yellow points), illustrating the ideal expression patterns of SDEG among many subtypes.

Consider four subtypes, ‘1’ denotes high expression, and ‘0’ denotes low expression. Then among all possible expression patterns in four subtypes, SDEGs are (0,0,0,1) (0,0,1,0) (0,1,0,0) (1,0,0,0). and non-SDEGs are (0,0,0,0) (1,1,1,1) (0,0,1,1) (0,1,0,1) (0,1,1,0) (1,0,0,1) (1,0,1,0) (1,1,0,0) (0,1,1,1) (1,0,1,1) (1,1,0,1) (1,1,1,0).

It can be seen that SDEG corresponds to the null hypothesis that $d_{j}=0$ in **Eq.2**, because every listed non-SDEGs (0,0,0,0) (1,1,1,1) (0,0,1,1) (0,1,0,1) (0,1,1,0) (1,0,0,1) (1,0,1,0) (1,1,0,0) (0,1,1,1) (1,0,1,1) (1,1,0,1) (1,1,1,0) satisfies $d_{j}=0$.

Please note that for detecting the SDEGs exclusively and lowly expressed in only one subtype but not in any others, i.e., (0,1,1,1) (1,0,1,1) (1,1,0,1) (1,1,1,0), $d_{j}$ can be defined as the gap between the subtypes expressed at the lowest and second lowest levels, and the same conclusion can be drawn.

Since ANOVA uses the null hypothesis focusing solely on (0,0,0,0) (1,1,1,1), i.e., the samples in all four subtypes are drawn from the same distribution, it will wrongly detect (0,0,1,1) (0,1,0,1) (0,1,1,0) (1,0,0,1) (1,0,1,0) (1,1,0,0) (0,1,1,1) (1,0,1,1) (1,1,0,1) (1,1,1,0) as SDEGs. In fact, these are false positive SDEGs because they are actually the subtype-nonspecific classic DEGs (differentially expressed across any of the subtypes).

## OVE-sFC test

### OVE-sFC test statistic

Motivated by our earlier work on OVE-FC^1^, we define One-Versus-Everyone Log Fold Change (OVE-LFC) as

$$\begin{aligned} d_{jk}=\mu_{k}\left( j \right)-\max_{l\neq k} \mu_{l}\left( j \right)=\min_{l\neq k} \left\{ \mu_{k}\left( j \right)-\mu_{l}\left( j \right) \right\}, k=1,\ldots,K,\#\left( S1 \right) \end{aligned}$$

where $\mu_{k}\left( j \right)$ is the mean of log2-transformed expressions of gene $j$ in subtype $k$. Conceptually, the null hypothesis for non-SDEGs of subtype $k$ and the alternative hypothesis for SDEGs of subtype$k$ can be described as

$$\begin{aligned} H_{SDEG\left( k \right)}^{null}:d_{jk}=0; \\ H_{SDEG\left( k \right)}^{alt}:d_{jk}>0;\#\left( S2 \right) \end{aligned}$$

It can be shown that SDEG corresponds to the above null hypothesis that $d_{j}$=0, because every expression pattern of non-SDEGs satisfies $d_{j}$=0. Standardizing OVE-LFC by within-subtype variance of gene$j$, we obtain the subtype-specificOVE-sFC test statistic

$$\begin{aligned} t_{jk}=\min_{l\neq k} \left\{ \frac{\mu_{k}\left( j \right)-\mu_{l}\left( j \right)}{\sigma(j)\sqrt{\frac{1}{N_{k}}+\frac{1}{N_{l}}}} \right\}=\min_{l\neq k} \left\{ t\text{-}stat_{k,l}\left( j \right) \right\}, k=1,\ldots,K,\#\left( S3 \right) \end{aligned}$$

where $\sigma^{2}(j)$ is the genewise variance of logarithmic expression within one subtype, ${t\text{-}stat}_{k,l}\left( j \right)$is t-statistic between subtype $k$ and $l$. Using $t_{jk}$ to select SDEGs for subtype$k$, denoted by $\mathrm{SDEG}(k)$, is equivalent to performing pairwise t-tests between subtype$k$ and each of the remaining subtypes and then taking their intersections, since the null hypothesis and alternative hypothesis can be rewritten as $H_{\mathrm{SDEG}\left( k \right)}^{null}=\bigvee H_{\mathrm{SDEG}\left( k,l \right)}^{null}$ and $H_{\mathrm{SDEG}\left( k \right)}^{alt}=\bigwedge H_{\mathrm{SDEG}\left( k,l \right)}^{alt}$ respectively, where $\mathrm{SDEG}(k,l)$ denotes SDEGs enriched in subtype$k$ against subtype$l$. However, naively combining the pairwise t-test p-values to assess the significance of SDEGs did not model the null distributions rigorously according to the definition of SDEGs.

Notice that we only need to compute $t_{jk}$ when $\mu_{k}\left( j \right)$ is the largest value among$\mu_{l}\left( j \right), l=1,\ldots,K$, as we only need to determine whether the highest expressed subtype has significant enrichment against all other subtypes. Thus, we can define the OVE-sFC test statistic, independent of any specific subtype, as

$$\begin{aligned} t_{j}=\max_{k=1,\ldots,K} \left\{ t_{jk} \right\}=t_{j\left( K \right)}\#\left( S4 \right) \end{aligned}$$

to test the significance level of gene $j$ as a SDEG (**Eq. 2 and Eq. 3**), where subscript $(K)$ indicates the $K$th order of ranked sequence $[\mu_{l}(j), l=1,\ldots,K]$, *i.e.,* the subtype with maximum mean.

### Assessing the statistical significance of candidate SDEGs

To evaluate the significance level of SDEGs, we need to estimate the distribution of the OV-sFC test statistics conditioned on the null hypothesis$H_{\mathrm{SDEG}}^{null}:d_{j}=0$, which includes the following $(K-1)$ the null hypotheses (**Fig. S1a**):

$$H_{\text{SDEG, }0}^{null}: \mu_{\left( K \right)}\left( j \right)=\mu_{\left( K-1 \right)}\left( j \right)=\ldots=\mu_{\left( 1 \right)}\left( j \right);$$

$$H_{\text{SDEG, 1}}^{null}: \mu_{\left( K \right)}\left( j \right)=\mu_{\left( K-1 \right)}\left( j \right)=\ldots=\mu_{\left( 2 \right)}\left( j \right)>\mu_{\left( 1 \right)}\left( j \right);$$

$$\ldots$$

$$H_{\text{SDEG, }K-2}^{null}: \mu_{\left( K \right)}\left( j \right)=\mu_{\left( K-1 \right)}\left( j \right)>\mu_{\left( K-2 \right)}\left( j \right), \ldots, \mu_{\left( 1 \right)}\left( j \right);$$

where subscript $(k)$ enclosed in parentheses indicates the $k$th order of $[\mu_{l}(j), l=1,\ldots,K]$. The null distribution of the OVE-sFC test statistic under $H_{\text{SDEG, }m}^{null},0<m\leq K-2$, is asymptotically equivalent to that under$H_{\text{SDEG, }m}^{null '}:\mu_{\left( K \right)}(j)=\ldots=\mu_{\left( m+1 \right)}(j)\gg\mu_{\left( m \right)}(j), \ldots, \mu_{\left( 1 \right)}(j)$ when $\Delta_{m}(j)=\mu_{\left( K \right)}(j)-\mu_{\left( m \right)}(j)$ is sufficiently large that only the highest expressed $(K-m)$ subtypes affect the OVE-sFC test statistics. Conversely, when $\Delta_{m}(j)$ approaches zero, $H_{\text{SDEG, }m}^{null}$ will tend to become $H_{\text{SDEG, }m-1}^{null}$ with the null distribution of the OVE-sFC test statistics becoming less dispersed, as shown in the following simulation.

To illustrate the change of the null distribution with varied $\Delta_{m}(j)$, we can calculate theoretical p-values based on a multivariate normal/student’s t-distribution of $(K-1)$ random variables ${t\text{-}stat}_{(K),l}, l=1,\ldots,K,l\neq(K)$ that have mean vector $[\boldsymbol{0}_{K-m-1},\Delta_{m}(j),\ldots,\Delta_{1}(j)]$, unit variances, and correlation coefficients

$$\begin{aligned} \rho_{l_{1},l_{2}}=\frac{1}{\sqrt{\left( \frac{N_{\left( K \right)}}{N_{l_{1}}}+1 \right)\left( \frac{N_{\left( K \right)}}{N_{l_{2}}}+1 \right)}}.\#\left( S5 \right) \end{aligned}$$

Decreasing $\Delta_{m}(j)$ tends to shift the distribution of $\min_{l\neq\left( K \right)} \left\{ {t\text{-}stat}_{(K),l} \right\}$ in the negative direction, increasing the significance level (smaller p-values). For example, when $K=3$ with an equal sample size for all subtypes, the correlation coefficient between $Z_{0}=t\text{-}stat_{\left( 3 \right),\left( 2 \right)}$ and $Z_{1}=t\text{-}stat_{\left( 3 \right),\left( 1 \right)}$ is $\rho=0.5$. Under the null hypothesis $H_{\text{SDEG, 1}}^{null}$, $Z_{0}$ has zero mean and $Z_{1}$ could have a positive mean. **Fig. S1b** shows the change of p-values with varying$Z_{1}$. If the mean of $Z_{1}$ is large, three-group OVE-sFC test statistics can be approximated by two-group t-statistics, yielding the same significance level as a t-test. Otherwise, the p-value will decrease and reach as low as 0.01387 when the mean of $Z_{1}$ also equals zero.

If we know both from which null hypothesis each gene comes and the true $\Delta_{m}(j)$, then we can calculate the theoretical p-value. Since these are unknown, they must be modeled and approximated. We propose a mixture model of the null distributions under the component hypotheses (**Eq. 4-5)** to estimate the p-values of candidate SDEGs (**Eq. 6-9**).

### Mixture model to assess statistical significance of candidate SDEGs

This subsection gives a detailed explanation of **Eq. 4-9**.

**(1) The order of subtypes**

When $H_{\text{SDEG, }m}^{null}$ is true, samples in the highest expressed$(K-m)$ subtypes are assumed to be drawn from the same populations. Hence, we must identify which $(K-m)$ subtypes are the most highly expressed. To obtain meaningful OVE-sFC test statistics (**Eq. 3**), we set the highest expressed subtype as being the subtype with the largest mean and sort the remaining subtypes by their t-statistics against the highest expressed subtype. The order of subtypes is exactly equal to the order of $[\mu_{l}(j), l=1,\ldots,K]$ when $N_{1}=\ldots=N_{K}$. The subtype with the largest estimated mean does not always have the largest true population mean, due to high variance, especially when sample sizes are unbalanced among all subtypes. Nonetheless, we can focus on the significance level of SDEGs for the subtype with the largest estimated mean. While a gene may have the highest expression in one of the remaining subtypes, and thus correspond to a more significant level in other subtypes, this gene is not likely to be an adequate marker with a low p-value.

**(2)** $\mathbf{f} \left\{ \boldsymbol{t} | \boldsymbol{H}_{\text{SDEG, }\boldsymbol{m}}^{\boldsymbol{null}} \right\}$

We approximate $H_{\text{SDEG, }m}^{null}$ by $H_{\text{SDEG, }m}^{null '}$ under which the null distribution is estimated by permuting sample labels in the top $(K-m)$ subtypes. These permutation distributions from different genes could be pooled together with certain weights where: **(a)** genes with large posterior probabilities of $H_{\text{SDEG, }m}^{null}$ should contribute more to the null distribution under $H_{\text{SDEG, }m}^{null}$ and **(b)** genes with relatively small $\Delta_{jm}$ may affect the null distribution estimation under both $H_{\text{SDEG, }m}^{null}$ and $H_{\text{SDEG, }m-1}^{null}$. Since the local FDR is a good estimator of the posterior probability of a the null hypothesis ^2^, the weight of each gene’s contribution to the null hypothesis is assigned according to the local FDR of the ANOVA of the observed expression values across certain subtypes (**Eq. 5**). Note that we include the genes in the $fdr_{\text{non-SDEG, m}}$ calculation with a probability of $\left\{ 1-\sum_{n=0}^{m-1} w_{\text{non-SDEG, }n}(j) \right\}$ so that the computation of $w_{\text{non-SDEG, }m}$ is less affected by genes associated with $H_{\text{SDEG, 0}}^{null},\ldots, H_{\text{SDEG, }m-1}^{null}$. R package “fdrtool” ^3^ is then used to estimate the local FDR. The null distribution of OVE-sFC test statistics under $H_{\text{SDEG, }m}^{null}$ are approximated by weighted permutations (**Eq. 7**).

**(3)** $\boldsymbol{P}\left\{ \boldsymbol{H}_{\text{SDEG, }\boldsymbol{m}}^{\boldsymbol{null}} | \boldsymbol{H}_{\text{SDEG}}^{\boldsymbol{null}} \right\}$

Given the posterior probabilities of all genes (**Eq. 5**), the component weights in the mixture null distribution are estimated directly by

$$\begin{aligned} P\left\{ H_{\text{SDEG, }m}^{null} | H_{\text{SDEG}}^{null} \right\}&=\frac{\Pr\left\{ H_{\text{SDEG, }m}^{null} \right\}}{\sum_{n=0}^{K-2} \Pr\left\{ H_{\text{SDEG, }n}^{null} \right\}} \\ &=\frac{\frac{1}{J}\sum_{j=1}^{J} \Pr\left\{ H_{\text{SDEG, }m}^{null} | \boldsymbol{s}\left( j \right) \right\}}{\frac{1}{J}\sum_{j=1}^{J} \sum_{n=0}^{K-2} \Pr\left\{ H_{\text{SDEG, }n}^{null} | \boldsymbol{s}\left( j \right) \right\}} \\ &=\frac{\sum_{j=1}^{J} w_{\text{non-SDEG, }m}\left( j \right)}{\sum_{j=1}^{J} \sum_{n=0}^{K-2} w_{\text{non-SDEG, }n}\left( j \right)},\#\left( S6 \right) \end{aligned}$$

*i.e.,* **Eq. 8**.

**(4) p-values of candidate SDEGs**

The final equation for OVE-sFC test p-values, **Eq. 9**, depends on $PJ(K-1)$ permutations and $J\left( K-1 \right)$ ANOVA tests. As the observations are also included as one random permutation, OVE-sFC test p-value has a lower bound of $\min_{j} \left\{ \sum_{m=0}^{K-2} w_{\text{non-SDEG, }m}\left( j \right) \right\}/P\sum_{m=0}^{K-2} \sum_{j=1}^{J} w_{\text{non-SDEG, }m}\left( j \right)$.

### Subtype-specific p-values of candidate SDEGs

Using $t_{j}=\max_{k=1,\ldots,K} \left\{ t_{jk} \right\}$ as a test statistic in K-group comparison is equivalent to using the absolute value of the statistic in the two-group differential test, providing a concise way to define extremes in the $K$ directions. However, if the test statistics have an asymmetric the null distribution for two sides in the two-group differential test, another widely-accepted choice is to double the smaller of the two tail regions^4, 5^. Extending this method to $K>2$ cases, we can calculate subtype-specific p-values based on the null distribution of each $t_{jk}, k=1,\ldots,K$, by

$$\begin{aligned} p\text{-}value\left( k \right)&=\Pr\left\{ T_{k}>t_{obs,k} | H_{\text{SDEG, }m}^{null} \right\} \\ &=\sum_{m=0}^{K-2} \Pr\left\{ T_{k}>t_{obs,k} | H_{\text{SDEG, }m}^{null} \right\}P\left\{ H_{\text{SDEG, }m}^{null} | H_{\text{SDEG}}^{null} \right\} \\ &=\frac{\sum_{m=0}^{K-2} \sum_{p=1}^{P} \sum_{j=1}^{J} w_{\text{non-SDEG, }m}\left( j \right)I\left( T_{jk,p}\geq t_{k,obs} \right)\text{ }}{P\sum_{m=0}^{K-2} \sum_{j=1}^{J} w_{\text{non-SDEG, }m}\left( j \right)},\#\left( S7 \right) \end{aligned}$$

as one-tailed p-values. We then multiply the smallest tail by K. $t_{k,obs}$ is the observed OVE-sFC test statistic for a gene being tested under subtype $k$, and $T_{jk,p}$ is the OVE-sFC test statistic in the $p$th permutation on $j$th gene under subtype $k$. We must also avoid the extra computational burden of calculating the OVE-sFC test statistic for every subtype. Thus, a gene’s p-value is calculated only for the subtype with the highest mean, $p\text{-}value((K))$, corresponding to a positive $t_{k,obs}$. The OVE-sFC test statistics of permutated samples, $T_{jk,p}$, are also calculated only for the subtype with the highest mean. The OVE-sFC test statistics for other subtypes must be negative and could be set to be any negative constant because $t_{k,obs}>T_{jk,p}$ always holds in this case. Then the multiple-tailed p-value becomes

$$\begin{aligned} p\text{-}value=\min_{k=1,\ldots,K} \left\{ p\text{-}value\left( k \right) \right\}*K≐p\text{-}value((K))*K.\#\left( S8 \right) \end{aligned}$$

The right-hand side of **Eq. S8** does not always hold, resulting in p-values > 1, which is also a drawback of tail-doubling for two-tailed p-values. However, p-values for subtypes other than $(K)$ are unimportant. Even if the smallest tail appears under other subtypes, this gene is less likely to be an adequate SDEG. “Multiplying a one-sided p-value by K” can also be explained as multiple testing correction. Each molecule is actually tested 𝐾 times but only the p-value associated with the highest expressed subtype is recorded.

Subtype-specific OVE-sFC test p-values differ from the overall p-values in **Eq. 9** when the OVE-sFC test statistic is asymmetric across subtypes due to unbalanced sample sizes and/or unbalanced null hypothesis compositions (structure). The null distribution of $H_{\text{SDEG, }m}^{null}, m>0,$ is varied for each subtype, either when sample sizes are unequal across subtypes (**Eq. S5)** or when the non-SDEGs are unevenly distributed in the scatter plot. One example for the latter case is that $m$ subtypes are closer to each other than to any others, causing these $m$ subtypes to have a larger conditional probability of $H_{\text{SDEG, }K-m}^{null}$.

### By-products of aggregating genewise posterior probability

#### **Estimating the conditional probability of one subtype being upregulated under the null hypothesis**

More subtypes will increase the complexity of the null hypotheses. $P\left\{ H_{\text{SDEG, }m}^{null} | H_{\text{SDEG}}^{null} \right\}, m=0,\ldots,K-2,$ only reflects the component weights of $(K-1)$ null hypothesis types, identifying no specific subtype. By aggregating the genewise posterior probabilities, we can obtain the probability of one subtype being upregulated conditioned on $H_{\text{SDEG}}^{null}$, which will affect the number of false positive SDEGs estimated for this subtype.

Suppose $H_{\text{SDEG, }m}^{null}, m=0,\ldots,K-2,$ has an equal prior probability for $(K-m)$ directions. Under the composition of multiple null hypotheses, we get an estimated probability of one subtype being upregulated under the null:

$$\begin{aligned} \Pr\left\{ up in k\left| H_{\text{SDEG}}^{null} \right. \right\}=\frac{\sum_{m=0}^{K-2} \sum_{j=1}^{J} w_{\text{non-SDEG, }m}\left( j \right)I\left( reOrder_{kj}\leq K-m \right)/(K-m)}{\sum_{m=0}^{K-2} \sum_{j=1}^{J} w_{\text{non-SDEG, }m}\left( j \right)}, \#\left( S9 \right) \end{aligned}$$

where $reOrder_{kj}$ is the reversed order of subtype $k$ sorted by expressions of gene $j$ across all subtypes. $I(*)$ indicates whether, for gene $j$, subtype $k$ is among the highest expressed$(K-m)$ subtypes and thus being upregulated with probability of $1/(K-m$).

#### **Landscape of gene expression patterns**

There are $\left( 2^{K}-1 \right)$ types of gene expression patterns in a real dataset: genes exclusively expressed in $1, 2, \ldots,$ or $K$ of subtypes. Genes that are undetected in any of subtypes have been eliminated during pre-processing. Aggregating the genewise posterior probabilities of the null hypotheses, $w_{\text{non-SDEG, }m}$, and of the alternative hypotheses, $w_{\text{SDEG}}\left( j \right)=1-\sum_{n=0}^{K-2} w_{\text{non-SDEG, }n}(j)$, can provide the landscape of complex expression patterns as

$$\begin{aligned} P\left\{ H_{\text{SDEG(A), }m}^{null} \right\}=\frac{1}{J}\sum_{j=1}^{J} w_{\text{non-SDEG, }m}\left( j \right)I\left( \left\{ k | reOrder_{kj}\leq K-m \right\}=A \right), \\ A\subseteq\left\{ 1,\ldots,K \right\}, \left| A \right|=K-m,0\leq m\leq K-2,\#\left( S10a \right) \\ \\ P\left\{ H_{\mathrm{SDEG}\left( k \right)} \right\}=\frac{1}{J}\sum_{j=1}^{J} w_{\text{SDEG}}\left( j \right)I\left( reOrder_{kj}=1 \right),1\leq k\leq K,\#\left( S10b \right) \end{aligned}$$

where $reOrder_{kj}$ is the reversed order of subtype $k$ sorted by expressions of gene $j$ across all subtypes. $A$could be any subset of $\left\{ 1,\ldots,K \right\}$ with $(K-m)$ elements. $\left\{ k | order_{kj}\leq K-m \right\}$ is a subset with elements being the indexes of $(K-m)$ highest expressed subtypes for gene $j$.

### Within-subtype variance estimator

OVE-sFC test adopts an empirical Bayes moderated variance estimator used in “limma” that leverages information across genes by assuming a conjugate prior distribution $\sigma^{2}(j)\sim\nu_{0}\sigma_{0}^{2}/\mathcal{X}_{\nu_{0}}^{2}$^6^. As $t\text{-}stat_{k,l}\left( j \right)$ with moderated variance estimator $\tilde{\sigma}_{j}^{2}$ follows a t-distribution on $\nu_{0}+N-K$ degrees of freedom, the increased degrees of freedom $\nu_{0}$ reflect the greater reliability associated with the smoothed variances. If a mean-variance relationship exists in expression data, *e.g.,* RNAseq data, “limma-voom” weights are incorporated into the linear modelling procedures to stabilize variance^7^. Other variance estimators designed for two-group differential analysis can also be easily modified and integrated into the multiple-group comparisons by OVE-sFC test. For example, ROTS ^8^ adds a constant to the pooled variance estimator to optimize reproducibility across bootstrap resampling’s.

### Relevant peer SDEG selection methods

Two OVR methods, OVR-FC and OVR t-test, are included in peer method comparison. Note the degrees of freedom in OVR t-test (Welch's t-test) vary across genes, generating different null distributions for each gene’s test. Therefore, ranking genes based on OVR t-stat is different from that based on OVR t-test p-values.

We only calculate OVR-FC, OVR t-stat, and OVR t-test p-values for the highest expressed subtype. We do the same for OVE-FC, OVE-sFC (**Eq.1**), and OVE-sFC test p-values (**Eq.9**) and subtype-specific t-test p-values (**Eq.S8**). We use a standard method to calculate p-values for the OVO t-test based on a pairwise differential test^9^:

$$\begin{aligned} p\text{-}value=\max_{l\neq(K)} \left\{ p_{(K)l} \right\}\#\left( S11 \right) \end{aligned}$$

where $p_{(K)l}$ is the p-value of differential test between subtype $(K)$ and subtype $l$. For a fair comparison between OVO t-test and OVE-sFC test, a moderated variance estimator “limma” is applied to pairwise differential analyses in OVO t-test. As the moderated variance estimator depends on the information across all genes for each subtype pair, all the $K(K-1)/2$ subtype pairs for all genes have to be tested. While OVO t-test leverages information from each subtype pair for variance modeling, OVE-sFC test leverages information from all subtypes. Moreover, OVE-sFC test re-estimates p-values by a novel permutation scheme to model the complex null distribution.

## Simulations and Evaluations

### Simulation study for validating OVE-sFC test statistics on type I error

We simulated a set of gene expression experiments with 10,000 genes, where the baseline expression levels are sampled from real microarray data of the purified replicates in GSE19380 ^10^. A portion of these genes are modeled as housekeeping genes under $H_{\text{SDEG, }0}^{null}$ that maintain their baseline expression values in/across all subtypes, e.g., the expression patterns of (0,0,0,0) ~ (1,1,1,1) illustrated in the toy example. The remaining genes under $H_{\text{SDEG, }m>0}^{null}$ are adjusted being upregulated to the same expression level in at least two subtypes depending upon *m* value, mimicking other types of non-SDEGs, e.g., the expression patterns of (0,0,1,1) (0,1,0,1) (0,1,1,0) (1,0,0,1) (1,0,1,0) (1,1,0,0) or (0,1,1,1) (1,0,1,1) (1,1,0,0), (1,1,1,0) illustrated in the toy example. The mean upregulation levels are drawn from a properly bounded uniform distribution $[1/K,1/(K-m)]$ in scatter space. For example, in the toy example of *K*=4, the uniform distribution of the assigned mean upregulation levels is [1/4, 1/2] for (0,0,1,1) (0,1,0,1) (0,1,1,0) (1,0,0,1) (1,0,1,0) (1,1,0,0), or [1/4, 1/3] for (0,1,1,1) (1,0,1,1) (1,1,0,0), (1,1,1,0). **Fig. 1** and **Fig. S1a** show the scatter space of a toy example with three subtypes, where black dash line corresponds to (0,1,1), (1,0,1) and (1,1,0) with upregulation levels drawn from uniform distribution [1/3, 1/2]. Please note that the assignment of the mean upregulation levels from a properly bounded uniform distribution in scatter space is solely for simulating the other while typical non-SDEGs that differ from the housekeeping genes under the classic/baseline null hypothesis. We shall acknowledge that, to assess the type 1 error rate by OVE-sFC under the null hypothesis, the assignment of the mean upregulation levels is not limited to only uniform distribution. Gene-wise variation was generated from an inverse chi-square distribution, $\sigma^{2}(j)\sim\nu_{0}\sigma_{0}^{2}/\mathcal{X}_{\nu_{0}}^{2}$, with the prior degree of freedom $\nu_{0}$ being 5 or 40 related to less or more stabilized variances, respectively. $\sigma_{0}$ is set to be 0.2, 0.5, or 0.8 to check the performance under different noisy scenarios. **Fig. 2a** and **Fig. S2** show type I error control in the setting of three subtypes with balanced/unbalanced sample sizes. While permuting all subtypes generates a less dispersed null distribution, and permuting only the top two subtypes results in a more compact distribution, our posterior weighted permutation scheme can achieve balance automatically. Moreover, when the percentage of housekeeping genes (*i.e.,* $H_{\text{SDEG, }0}^{null}$) increases, the null distribution of OVE-sFC test statistics tends to be that generated from permuting all three subtypes. Conversely, those calculated by permuting the top two subtypes approximate true p-values in $H_{\text{SDEG, }1}^{null}\text{-}$dominated experiments. Comparison of results with different $\sigma_{0}$ shows that our test can control the type I error rate even in very noisy scenarios. However, small $\nu_{0}$ generates a modestly inflated type I error control, especially when sample sizes are small and the true $H_{\text{SDEG, }1}^{null}$ dominates. This effect may be due to a less reliable estimation of the moderated variance caused by small $\nu_{0}$ and sample sizes.

The estimated conditional probabilities of $H_{\text{SDEG, }0}^{null}$, $P\left\{ H_{\text{SDEG, }0}^{null} | H_{\text{SDEG}}^{null} \right\}$ in our model are expected to match the true proportions of housekeeping genes. As seen in **Fig. S3**, in noisy scenarios with high $\sigma_{0}$, $P\left\{ H_{\text{SDEG, }0}^{null} | H_{\text{SDEG}}^{null} \right\}$ tends to get over-estimated, implying that many genes sampled from the true $H_{\text{SDEG, }1}^{null}$ are treated as being from $H_{\text{SDEG, }0}^{null}$ in certain weights. These true $H_{\text{SDEG, }1}^{null}$ genes have no significantly large $\Delta_{1}(j)$, which is the difference between the top two subtypes and the third subtype. Thus, the null distributions generated from these genes are expected to be intermediate between those from true $H_{\text{SDEG, }0}^{null}$ (permuting three subtypes) and from true $H_{\text{SDEG,} 1}^{null '}$ (permuting top 2 subtypes).

Subtype-specific OVE-sFC test conducts the tests for SDEGs of each subtype separately, with the benefit that all subtypes exhibit a broadly similar type I error rate (**Fig. 2a**). Otherwise, a subtype with a smaller sample size will contribute more false positive SDEGs. Note that subtype-specific p-values could be larger than 1 after multiple-tail scaling (**Fig. 2b**) and so are truncated at 1. While we only calculate the p-value for the subtype with the largest group mean to reduce the computational burden, we ignore the possibility that genes with large p-values (around one) could have smaller p-values associated with another subtype. Since these genes are not likely to be valid SDEGs, it is not necessary to provide their precise p-values.

All the above analyses are repeated for SDEG identification involving five subtypes, producing p-values that control error rates correctly over a wide range of simulation scenarios (**Fig. 3a**). Since we randomly assigned genes under $H_{\text{SDEG, }m}^{null}, m>0$ without considering subtypes in the simulations above, the null hypothesis compositions are almost the same for all subtypes. To check the type I error rate with unbalanced null hypothesis compositions, five baseline profiles are generated by simulating two cell lines but assigning one to two subtypes and the other to three subtypes. Hence, the first cell line’s up-regulated genes become two subtypes’ $H_{\text{SDEG, }3}^{null}$ genes, while the second cell line’s up-regulated genes become three subtypes’ $H_{\text{SDEG, }2}^{null}$ genes. No true SDEGs exist for any of the five subtypes. OVE-sFC test (overall or subtype-specific) controls type I error rates well but identifies more false positive SDEGs in the first two subtypes (**Fig. 3b**). The unbalance of the null hypotheses leads to different probability estimates for a specific subtype being upregulated (**Eq. S9**). As simulated up-regulated genes in two cell lines are the same but are divided into two or three subtypes evenly, the first two subtypes have a higher probability of being upregulated, thus more false positive SDEGs are expected (**Fig. S4**). In our simulations, when the ratio of housekeeping genes is high, the unbalance becomes slight and thus approximately 1/5 of false positive SDEGs are allocated to each subtype. Conversely, a low ratio of housekeeping genes intensifies the uneven distribution of non-SDEGs in the scatter plot, increasing the number of false positive SDEGs detected in the first two subtypes. Subtype-specific tests can reduce this impact.

### Simulation study for assessing the power of OVE-sFC test statistics

A well-designed test can maximize power while controlling FDR below the expected level. In data sets containing true SDEGs, we evaluated several SDEG selection methods with respect to FDR control and pAUC. We studied whether the tests can control the FDR well by checking whether the true FDR with q-value (estimated by ‘fdrtool’ package ^3^) at 0.05 cutoff is also around 0.05. The area under the receiver operating characteristic (ROC) curve reflects whether the methods are able to rank true SDEGs above true non-SDEGs. In practical analyses, we emphasize the sensitivity or power of SDEG detection methods when the False Positive Rate (FPR) is significantly low, *e.g.,* 0.05/0.01 cutoff. Hence, the partial area under curve (pAUC) with specificity larger than 0.95/0.99 (equivalent to FPR less than 0.05/0.01) was used to evaluate the power of each method. Also, the emergence of mismatched detections (True Positive but associated with incorrect subtypes) with low specificity may exhibit a false high power.

Simulation settings are similar to the above subsection except that a portion of genes are designated as SDEGs with upregulation in one of the subtypes. We conducted three simulation sets to assess the power of OVE-FC/sFC test statistics and peer methods: (1) microarray simulations with variance drawn from an inverse chi-square distribution ($K$=3); (2) microarray simulations with variance sampled from real data ($K$=7); (3) RNASeq simulations with variance sampled from real data ($K$=7). The latter two simulation sets are more challenging with more subtypes and noisy RNASeq data, which help to show the benefit of using all subtypes for modeling by the proposed OVE-sFC test.

In the first simulation set, genewise variation was drawn from $\sigma^{2}\left( j \right)\sim\frac{\nu_{0}\sigma_{0}^{2}}{\mathcal{X}_{\nu_{0}}^{2}}$, with the prior degree of freedom $\nu_{0}$ being 5 or 40 related to less or more stabilized variances, respectively. $\sigma_{0}$ is set to be 0.2, 0.5, or 0.8 to check the performance under different noisy scenarios. 20% of genes are upregulated in one subtype with fold change following a uniform distribution in the Ternary plot (**Fig. S1a**), among which fold change ranges from 1 to $+\infty$. SDEGs with small fold change (close to 1) are non-ideal SDEGs. 40% of the remaining genes are under $H_{\text{SDEG, }0}^{null}$ and 40% under $H_{\text{SDEG, }1}^{null}$. Two scenarios were tested with unbalanced sample sizes or unbalanced null hypothesis compositions. In the former, sample size in each subtype is 3, 6, and 9, respectively, while $H_{\text{SDEG, }1}^{null}$ genes are distributed evenly. In the latter, each subtype has three samples and $H_{\text{SDEG, }1}^{null}$ genes only appear in the first two subtypes, which is a common case in real data: two subtypes are closer to each other than to the other subtypes. Each simulation was repeated 20 times. Both the overall and subtype-specific OVE-sFC tests could control FDR around the expected 0.05 level at q-value cutoff 0.05, with modestly weaker control in the case of small $\nu_{0}$ (**Fig. S5**). OVR t-test is too liberal, while OVO t-test is too conservative. Furthermore, a subtype-specific test can alleviate the unbalance of false positive SDEGs among subtypes. Other peer methods detected more false positive SDEGs in subtypes with a small sample size or with a large probability of being upregulated under $H_{\text{SDEG}}^{null}$ (**Eq. S9**). In terms of pAUC, OVE-sFC, OVE-FC and OVO t-test achieved better performance than OVR methods (**Table S1**). OVE-sFC outperforms OVE-FC due to the many non-ideal SDEGs present in this simulation set.

In the second and third simulation sets, genewise variation was sampled from microarray data GSE28490 or RNAseq data GSE60424. To keep the potential mean-variance trend, we divided genes from real data into 100 buckets based on the logarithm of their expression means and randomly selected a real variance for each simulated gene from the bucket that contained the simulated expression mean. 100 SDEGs were simulated for each subtype while the remaining genes were simulated as different yet realistic types of non-SDEGs, $H_{\text{SDEG, }m}^{null},m=0,1,\ldots,5$. The null hypothesis structure, *i.e.,* the percentage of different types of non-SDEGs for each subtype, was obtained from the estimated probability of gene expression patterns (**Eq. S10a**) from real data. Since this the null hypothesis structure from real data is somewhat unbalanced, we also simulated a balanced null hypothesis structure, where each subtype has the same percentage of different types of non-SDEGs and the sample size is either balanced or unbalanced. To compare the performance of detecting ideal/strict SDEGs (significantly large fold change) or detecting more realistic SDEGs (sufficiently large fold change), the fold changes of simulated SDEGs were drawn from a uniform distribution with different range: [2,20], [5,20], or [10,20]. Both the overall and subtype-specific OVE-sFC tests control the FDR around the expected 0.05 level at q-value cutoff 0.05, with slightly weaker control for noisy RNASeq data with a small sample size (**Fig. S6**). In terms of pAUC, OVE-sFC and OVE-FC approach the highest power in detecting true SDEGs (**Table S2-S3**, **Fig. 4**, **and Fig. S7-S8**).

### Real datasets to detect human immune cell markers

We used two real microarray datasets, GSE28490 (Roche) and GSE28491 (HUG), which contain mRNA expression profiles of seven immune cell types (B cells, CD4+ T cells, CD8+ T cells, NK cells, monocytes, neutrophils, and eosinophils) isolated from healthy human blood pools ^11^. Two datasets used the same protocols for cell isolation and sample processing on two independent panels of donors. All cell types have five samples in each dataset except for monocytes (ten samples in the Roche dataset). Cell types with few samples (2 neutrophils in Roche, 1 eosinophil in Roche, 2 eosinophils in HUG) were removed from datasets as outliers (**Table S7**). After eliminating low expressed probesets with an average log_2_ RMA signal value > 6 in none of the cell type groups, 12022/11339 probesets in Roche/HUG datasets respectively were considered to be expressed above background and thus available for use with OVE-sFC test.

### Evaluate SDEGs by supervised deconvolution performance

#### **Supervised deconvolution by CAM score**

There are two major classes of supervised deconvolution approaches to quantify proportions of subtypes in mixtures: fitting coefficients for the linearly modeled relationship between mixture and pure expression levels, or scoring each subtype by expression levels of markers in mixtures^12^. The former approach estimates the absolute fraction of each subtype in a heterogeneous sample. The latter approach provides relative scores that are comparable across samples but not between subtypes. However, the second class has some advantages. **(1)** It only assumes the indexes of markers are unchangeable, so that the markers selected based on pure expression levels can help deconvolute mixtures in different microenvironments, even when measured on other platforms. **(2)** Scores are computed for each subtype individually and thus less affected by each other and unknown subtypes. **(3)** Scores are able to achieve a higher correlation with ground truth proportions, since they focus on catching the dynamic trend across samples, not the absolute proportion values. Generally, the factors affecting coefficient fitting are complex, whereas scoring one subtype relies mostly on the precision of the indexes of selected markers. Therefore, we adopted a scoring approach for supervised deconvolution and use its performance to evaluate selected markers. Details of the score estimation are presented below.

The expression level of the $j$th gene in the $i$th mixture sample, $x_{ij}$, is modeled as a linear combination of the expression levels of that gene across the $K$ subtypes present in the mixture, weighted by their constituent proportions $a_{ik}$:

$$\begin{aligned} x_{ij}=\sum_{k=1}^{K} a_{ik}s_{k}\left( j \right).\#\left( S12 \right) \end{aligned}$$

Consider a set of N heterogeneous samples and denote $x\left( j \right)=\left[ x_{1j},\ldots,x_{Nj} \right], a_{k}=[a_{1k},\ldots,a_{Nk}]$. We can re-write **Eq. S12** as

$$\begin{aligned} x\left( j \right)=\sum_{k=1}^{K} s_{k}\left( j \right)a_{k}.\#\left( S13 \right) \end{aligned}$$

As molecular markers are exclusively expressed in only one of each subtype, we have

$$\begin{aligned} x\left( j_{MG\text{-}k} \right)\approx s_{k}\left( j_{MG\text{-}k} \right)a_{k},\#\left( S14 \right) \end{aligned}$$

where $j_{MG\text{-}k}$ is the index of any marker of subtype $k$. Therefore, the expression levels of markers are proportional to the constituent fraction of certain subtypes. After applying a sum-to-one standardization to $x(j_{MG\text{-}k})$, their space median can be used as the score for subtype $k$:

$$\begin{aligned} \tilde{a}_{k}=SpaceMedian\left\{ \tilde{x}\left( j_{MG\text{-}k} \right) \right\}.\#\left( S15 \right) \end{aligned}$$

“l1median” function in R package “pcaPP”^13^ is used to find space median.

Such score estimation approach is the same as part of an unsupervised deconvolution algorithm - “CAM” ^14^. We can call **Eq. S15** a CAM score. There are also other score methods among which MCP-counter score ^15^, the geometric mean of $x\left( j_{MG\text{-}k} \right)$, is the most similar one to CAM score. We expect the space median operation to enhance the robustness of scores. Sample-specific enrichment score, e.g. ssGSEA, has also been proposed to infer subtypes in tissues ^16, 17^, but its relationship with the true subtype proportion is non-linear and unclear.

#### ***In silico* simulated mixtures**

We generated gene expression readouts of *in silico* heterogeneous samples following the linear mixing model in **Eq. S12** with additional biological variation and technical variation:

$$\begin{aligned} x_{ij}=\sum_{k=1}^{K} a_{ik}\left( s_{k}\left( j \right)+\Delta s_{ik}\left( j \right) \right)+\varepsilon_{ij},\#\left( S16 \right) \end{aligned}$$

where $\Delta s_{ik}\left( j \right)$ reflects subtype-specific biological variation across samples and $\varepsilon_{ij}$ is technical noise of measurement. In the first and the third simulation (**Fig. 6a, 6c**), $s_{k}(j)$ is cell-type specific mRNA expression mean (averaged across samples in log2 scale and transformed to original scale) in GSE28491. $\Delta s_{ik}\left( j \right)$ and $\varepsilon_{ij}$ follows zero-mean Gaussian distribution, with variance drawn from the inverse chi-square distribution with $\sigma_{0}$ being 0.5 and 0.1, $\nu_{0}$ being 10 and 5, respectively. In the second simulation (**Fig. 6b**), as GSE60424 has almost 20 samples per cell type, one sample was randomly selected from each cell type and its RNAseq count profile treated as $s_{k}\left( j \right)$ with biological and technical variance already included. Mixing proportions, $a_{ik},k=1,\ldots,K$, were drawn randomly from a flat Dirichlet distribution and used in the three simulations.

## Supplementary Tables

**Table S1** pAUC (FPR<0.05) obtained from Microarray simulations involving 3 subtypes and with various experimental settings

|  |  | $\nu0=5$ | | | $\nu0=40$ | | |
| --- | --- | --- | --- | --- | --- | --- | --- |
|  |  | $\sigma=0.2$ | $\sigma=0.5$ | $\sigma=0.8$ | $\sigma=0.2$ | $\sigma=0.5$ | $\sigma=0.8$ |
| Balanced  The null  Hypothesis  Structure  Unbalanced  Sample  Size  (n=3,6,9) | ANOVA | 0.53747 | 0.5402 | 0.54266 | 0.53794 | 0.54081 | 0.54344 |
|  | OVR-FC | 0.67778 | 0.65644 | 0.62664 | 0.68155 | 0.66657 | 0.64723 |
|  | OVR t-stat | 0.9093 | 0.77705 | 0.6904 | 0.92751 | 0.80715 | 0.71382 |
|  | OVR t-test | 0.82649 | 0.70774 | 0.64513 | 0.84036 | 0.73137 | 0.66212 |
|  | OVO t-test | 0.94982 | 0.8569 | 0.76983 | 0.96193 | 0.89067 | 0.80318 |
|  | OVE-FC | 0.94292 | 0.84033 | 0.74264 | 0.96179 | 0.88991 | 0.80195 |
|  | OVE-sFC | **0.95116** | **0.85907** | **0.77208** | **0.96196** | **0.89074** | **0.80403** |
|  | sub OVE-sFC | **0.95119** | **0.85915** | **0.77301** | **0.96211** | **0.89078** | **0.80424** |
| Unbalanced  The null  Hypothesis  Structure  (n=3,3,3) | ANOVA | 0.54128 | 0.54473 | 0.54533 | 0.54105 | 0.54472 | 0.54411 |
|  | OVR-FC | 0.70822 | 0.67034 | 0.62272 | 0.71086 | 0.68668 | 0.65558 |
|  | OVR t-stat | 0.92793 | 0.78802 | 0.68392 | 0.94068 | 0.81609 | 0.70628 |
|  | OVR t-test | 0.89682 | 0.73482 | 0.6429 | 0.91385 | 0.75827 | 0.65584 |
|  | OVO t-test | 0.93685 | 0.8218 | 0.72267 | 0.95218 | 0.86223 | 0.76863 |
|  | OVE-FC | 0.93113 | 0.80302 | 0.69664 | 0.9522 | 0.86167 | 0.7676 |
|  | OVE-sFC | **0.93887** | **0.82354** | **0.72451** | **0.95211** | **0.86229** | **0.76811** |
|  | sub OVE-sFC | **0.94075** | **0.82749** | **0.72821** | **0.95383** | **0.86651** | **0.77229** |

**Table S2** pAUC (FPR<0.05 and 0.01) obtained from Microarray simulations involving 7 subtypes and with various experimental settings

|  |  | pAUC (FPR<0.05) | | | pAUC (FPR<0.01) | | |
| --- | --- | --- | --- | --- | --- | --- | --- |
|  |  | $FC\in[2,20]$ | $FC\in[5,20]$ | $FC\in[10,20]$ | $FC\in[2,20]$ | $FC\in[5,20]$ | $FC\in[10,20]$ |
| Unbalanced  The null  Hypothesis  Structure  3 samples  /per subtype | ANOVA | NA | NA | NA | NA | NA | NA |
|  | OVR-FC | NA | 0.50997 | 0.56310 | NA | NA | 0.50035 |
|  | OVR t-stat | 0.81362 | 0.92408 | 0.96226 | 0.80365 | 0.91644 | 0.95595 |
|  | OVR t-test | 0.55572 | 0.57203 | 0.59205 | 0.56113 | 0.57531 | 0.59397 |
|  | OVO t-test | 0.93403 | 0.98283 | 0.98955 | 0.89324 | 0.96797 | 0.97626 |
|  | OVE-FC | 0.93612 | **0.99533** | **0.99878** | 0.81569 | **0.98423** | **0.99759** |
|  | OVE-sFC | **0.94797** | 0.98833 | 0.99348 | **0.92393** | 0.97953 | 0.98765 |
|  | sub OVE-sFC | **0.95052** | 0.98897 | 0.99384 | **0.92372** | 0.98028 | 0.98764 |
| Balanced  The null  Hypothesis  Structure  3 samples  /per subtype | ANOVA | NA | NA | NA | NA | NA | NA |
|  | OVR-FC | NA | 0.50671 | 0.55154 | NA | NA | 0.50275 |
|  | OVR t-stat | 0.82253 | 0.92019 | 0.94936 | 0.80918 | 0.91560 | 0.94403 |
|  | OVR t-test | 0.56269 | 0.58333 | 0.58408 | 0.56490 | 0.58558 | 0.58595 |
|  | OVO t-test | 0.92870 | 0.98165 | 0.98961 | 0.88065 | 0.96383 | 0.97822 |
|  | OVE-FC | 0.93763 | **0.99568** | **0.99770** | 0.82500 | **0.98386** | **0.98897** |
|  | OVE-sFC | **0.95044** | 0.98929 | 0.99317 | **0.91775** | 0.98127 | **0.98948** |
|  | sub OVE-sFC | **0.95062** | 0.98935 | 0.99316 | **0.91830** | 0.98163 | **0.98947** |
| Balanced  The null  Hypothesis  Structure  Unbalanced  Sample  Size  (3,3,3,4,5,5,5) | ANOVA | NA | NA | NA | NA | NA | NA |
|  | OVR-FC | NA | 0.50686 | 0.54375 | NA | NA | 0.50004 |
|  | OVR t-stat | 0.79462 | 0.90719 | 0.94392 | 0.78328 | 0.89854 | 0.93593 |
|  | OVR t-test | 0.52553 | 0.54197 | 0.55172 | 0.52574 | 0.54076 | 0.55029 |
|  | OVO t-test | 0.95338 | 0.99001 | 0.99563 | 0.92252 | 0.98104 | 0.98687 |
|  | OVE-FC | 0.95395 | **0.99763** | **0.99882** | 0.85616 | **0.98841** | **0.99423** |
|  | OVE-sFC test | **0.96077** | 0.99411 | 0.99711 | **0.94228** | 0.98681 | 0.99301 |
|  | sub OVE-sFC | **0.96101** | 0.99418 | 0.99711 | **0.94222** | 0.98693 | 0.99317 |

**Table S3** pAUC (FPR<0.05 and 0.01) obtained from RNAseq simulations involving 7 subtypes and with various experimental settings

|  |  | pAUC (FPR<0.05) | | | pAUC (FPR<0.01) | | |
| --- | --- | --- | --- | --- | --- | --- | --- |
|  |  | $FC\in[2,20]$ | $FC\in[5,20]$ | $FC\in[10,20]$ | $FC\in[2,20]$ | $FC\in[5,20]$ | $FC\in[10,20]$ |
| Unbalanced  The null  Hypothesis  Structure  3 samples  /per subtype | ANOVA | NA | NA | NA | NA | NA | NA |
|  | OVR-FC | 0.50007 | 0.51649 | 0.59034 | 0.50120 | 0.50260 | 0.52331 |
|  | OVR t-stat | 0.63528 | 0.76419 | 0.81669 | 0.60403 | 0.70409 | 0.77145 |
|  | OVR t-test | 0.54461 | 0.60287 | 0.63803 | 0.53923 | 0.58136 | 0.62174 |
|  | OVO t-test | 0.77600 | 0.88615 | 0.93747 | 0.68080 | 0.76939 | 0.85403 |
|  | OVE-FC | 0.75622 | **0.93221** | **0.97183** | 0.62926 | 0.80988 | **0.90791** |
|  | OVE-sFC | **0.79344** | 0.91728 | 0.96178 | **0.68795** | **0.81828** | 0.89212 |
|  | sub OVE-sFC | **0.79869** | 0.91810 | 0.96212 | **0.69353** | **0.81877** | 0.89454 |
| Unbalanced  The null  Hypotheses  20 samples  /per subtype | ANOVA | NA | NA | NA | NA | NA | NA |
|  | OVR-FC | NA | 0.53594 | 0.62450 | NA | 0.50883 | 0.53201 |
|  | OVR t-stat | 0.61767 | 0.75120 | 0.82295 | 0.60970 | 0.72330 | 0.80238 |
|  | OVR t-test | 0.50731 | 0.55117 | 0.60501 | 0.51059 | 0.54524 | 0.59214 |
|  | OVO t-test | 0.97075 | 0.99193 | 0.99578 | **0.96192** | 0.98840 | 0.99354 |
|  | OVE-FC | **0.97611** | 0.99369 | 0.99660 | 0.95103 | 0.98974 | 0.99263 |
|  | OVE-sFC | 0.97232 | **0.99448** | **0.99782** | 0.95100 | **0.99131** | **0.99632** |
|  | sub OVE-sFC | 0.97363 | **0.99438** | **0.99770** | 0.95323 | **0.99093** | **0.99645** |
| Balanced  The null  Hypothesis  Structure  3 samples  /per subtype | ANOVA | NA | NA | NA | NA | NA | NA |
|  | OVR-FC | NA | 0.52764 | 0.59584 | NA | 0.50463 | 0.52588 |
|  | OVR t-stat | 0.63400 | 0.76959 | 0.82773 | 0.60631 | 0.72285 | 0.77652 |
|  | OVR t-test | 0.54489 | 0.61288 | 0.63604 | 0.54151 | 0.59807 | 0.62051 |
|  | OVO t-test | 0.77225 | 0.89831 | 0.93346 | 0.67555 | 0.79982 | 0.86136 |
|  | OVE-FC | 0.74739 | **0.92323** | **0.97642** | 0.61980 | 0.78167 | **0.91866** |
|  | OVE-sFC | **0.77921** | 0.91873 | 0.96874 | **0.68426** | **0.81631** | 0.91196 |
|  | sub OVE-sFC | **0.77931** | 0.91857 | 0.96867 | **0.68397** | **0.81483** | 0.91164 |
| Balanced  The null  Hypothesis  Structure  Unbalanced  Sample  Size  (3,3,3,4,5,5,5) | ANOVA | NA | NA | NA | NA | NA | NA |
|  | OVR-FC | NA | 0.51603 | 0.57669 | NA | 0.50377 | 0.51511 |
|  | OVR t-stat | 0.61345 | 0.74834 | 0.79636 | 0.59512 | 0.70675 | 0.76168 |
|  | OVR t-test | 0.53701 | 0.58239 | 0.62472 | 0.53539 | 0.56349 | 0.60675 |
|  | OVO t-test | 0.79078 | 0.91174 | 0.93552 | 0.69959 | 0.82953 | 0.87458 |
|  | OVE-FC | 0.75689 | **0.95120** | **0.97319** | 0.61997 | **0.85831** | **0.92000** |
|  | OVE-sFC | **0.79951** | 0.92714 | 0.95949 | **0.70584** | 0.84436 | 0.89120 |
|  | sub OVE-sFC | **0.79978** | 0.92689 | 0.95925 | **0.70544** | 0.84413 | 0.88982 |

**Table S4** Counts of cell-type specific markers under certain threshold

| Threshold | Subtype | Measured only  in Roche | Markers only  in Roche | Markers  in both | Markers only  in HUG | Measured only  in HUG | Total |
| --- | --- | --- | --- | --- | --- | --- | --- |
| q-value  <0.05 | B cells | 70 | 266 | 474 | 528 | 39 | 1377 |
|  | CD4+ T cells | 6 | 44 | 28 | 54 | 1 | 133 |
|  | CD8+ T cells | 8 | 55 | 7 | 33 | 2 | 105 |
|  | NK cells | 83 | 563 | 208 | 68 | 13 | 935 |
|  | Eosinophils | 37 | 301 | 204 | 106 | 11 | 659 |
|  | Monocytes | 51 | 475 | 630 | 463 | 55 | 1674 |
|  | Neutrophils | 43 | 519 | 626 | 256 | 76 | 1520 |
| q-value  <0.001 | B cells | 56 | 209 | 264 | 102 | 13 | 644 |
|  | CD4+ T cells | 2 | 12 | 4 | 4 | 0 | 22 |
|  | CD8+ T cells | 3 | 20 | 3 | 1 | 0 | 27 |
|  | NK cells | 52 | 255 | 85 | 8 | 4 | 404 |
|  | Eosinophils | 27 | 208 | 55 | 13 | 4 | 307 |
|  | Monocytes | 46 | 427 | 260 | 67 | 18 | 818 |
|  | Neutrophils | 24 | 452 | 173 | 31 | 32 | 712 |
| Corrected  p-value  <0.05 | B cells | 44 | 134 | 181 | 55 | 9 | 423 |
|  | CD4+ T cells | 1 | 4 | 2 | 0 | 0 | 7 |
|  | CD8+ T cells | 0 | 14 | 1 | 0 | 0 | 15 |
|  | NK cells | 40 | 185 | 37 | 1 | 3 | 266 |
|  | Eosinophils | 19 | 49 | 24 | 15 | 0 | 107 |
|  | Monocytes | 36 | 289 | 137 | 20 | 5 | 487 |
|  | Neutrophils | 13 | 203 | 56 | 13 | 15 | 300 |
| Corrected  p-value  <0.001 | B cells | 41 | 116 | 148 | 44 | 9 | 358 |
|  | CD4+ T cells | 1 | 5 | 1 | 0 | 0 | 7 |
|  | CD8+ T cells | 0 | 13 | 1 | 0 | 0 | 14 |
|  | NK cells | 32 | 158 | 18 | 2 | 3 | 213 |
|  | Eosinophils | 19 | 61 | 12 | 2 | 0 | 94 |
|  | Monocytes | 33 | 296 | 60 | 1 | 0 | 390 |
|  | Neutrophils | 13 | 255 | 3 | 1 | 3 | 275 |

**Table S5** Statistics of CD4+ T cell markers detected in both Roche and HUG (q<0.05)

**Table S6** Statistics of CD8+ T cell markers detected in both Roche and HUG (q<0.05)

**Table S7** Sample size of each cell type in four datasets

|  | GSE28490 | GSE28491 | GSE60424 | GSE72056* |
| --- | --- | --- | --- | --- |
| B cells | 5 | 5 | 20 | 628 |
| CD4+ T cells | 5 | 5 | 20 | 873 |
| CD8+ T cells | 5 | 5 | 20 | 1099 |
| Regulatory T cells | NA | NA | NA | 141 |
| NK cells | 5 | 5 | 14 | 89 |
| Eosinophils | 4 | 3 | NA | NA |
| Macrophages/Monocytes | 10 | 5 | 20 | 170 |
| Neutrophils | 3 | 5 | 20 | NA |
| Plasmacytoid dendritic cells | 5 | NA | NA | 26 |
| Myeloid dendritic cells | 5 | NA | NA | NA |
| Endothelial cells | NA | NA | NA | 71 |
| Cancer associated fibroblasts | NA | NA | NA | 96 |

*Cell type labels are from single-cell classification results ^18^

## Supplementary Figure

**
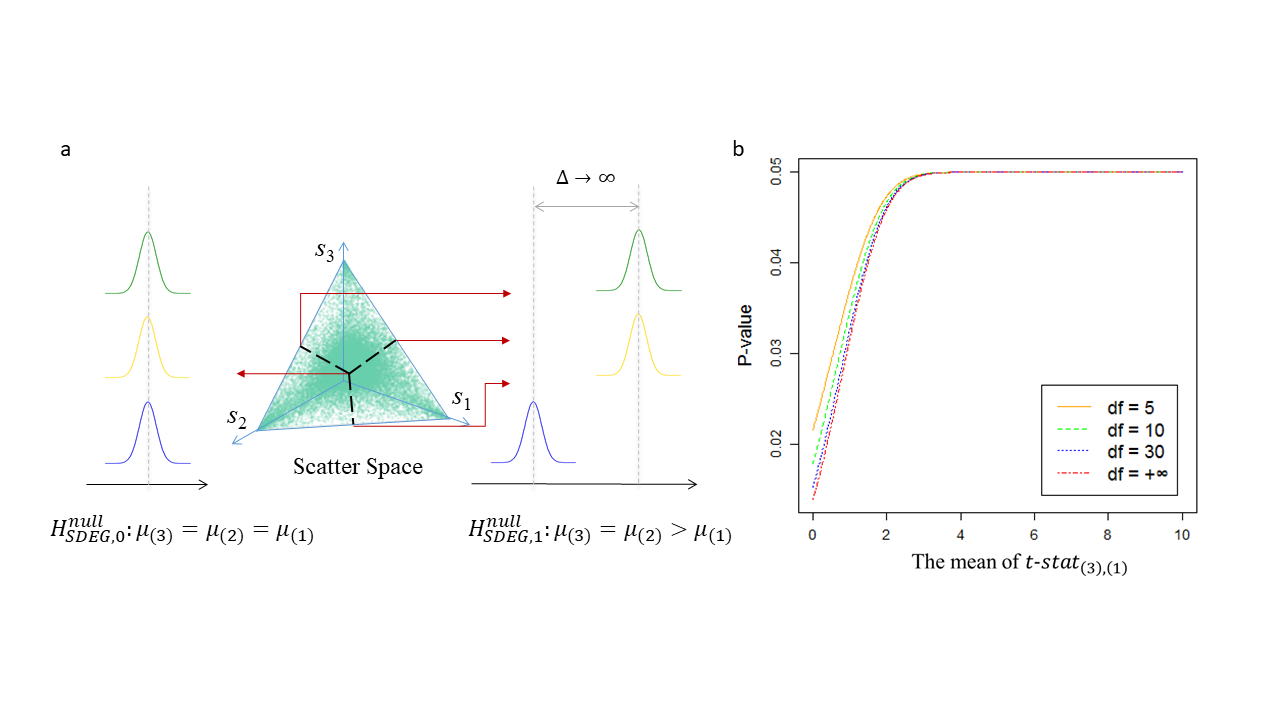
**

**Figure S1.** Two extreme cases of non-SDEGs when the subtype number is three. Fixing the critical value to be that of two-group t-test p = 0.05, p-values approach the upper bound when two subtypes are drawn from the same population with much larger expressions than the third. On the contrary, p-values decrease to be minimum when all three subtypes are drawn from the same population.

**
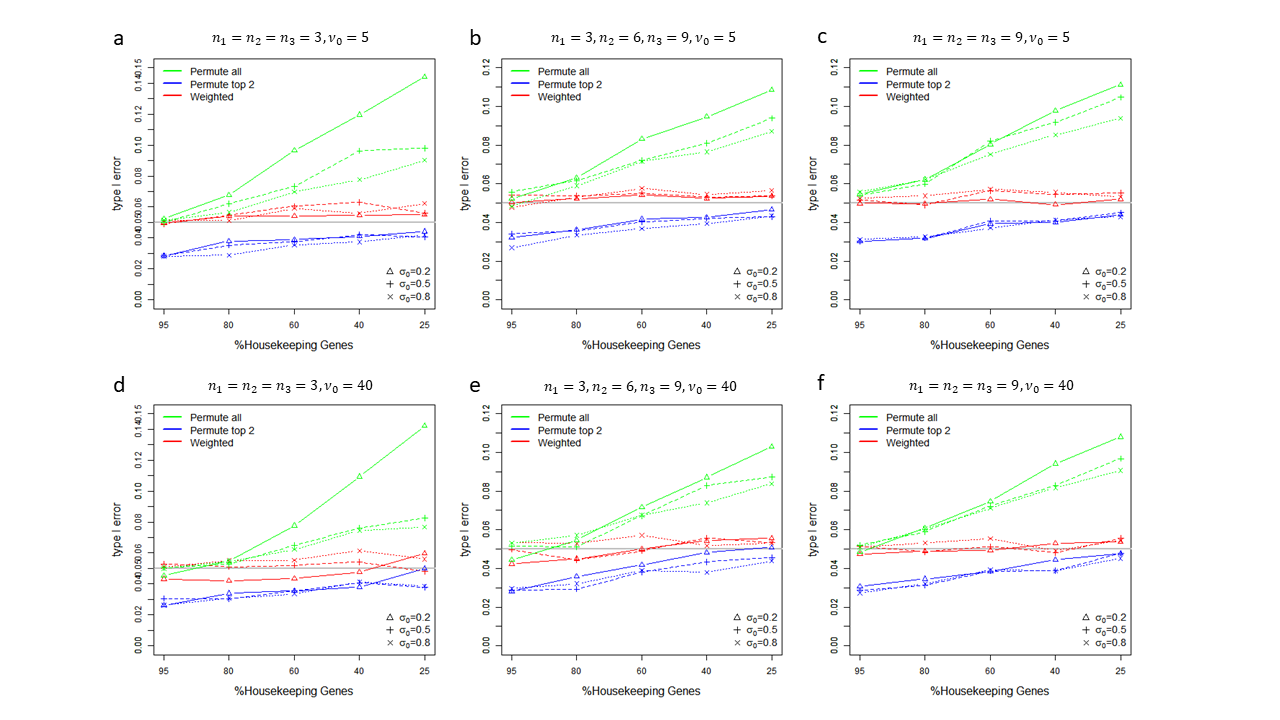
**

**Figure S2.** Comparisons of type I error rates from different permutation schemes under different settings of noisy scenarios and sample sizes (housekeeping genes: 95%, 80%, 60%, 40%, or 25%; $\sigma_{0}$: 0.2, 0.5, or 0.8; $\nu_{0}$: 5(top), or 40(bottom)).

**
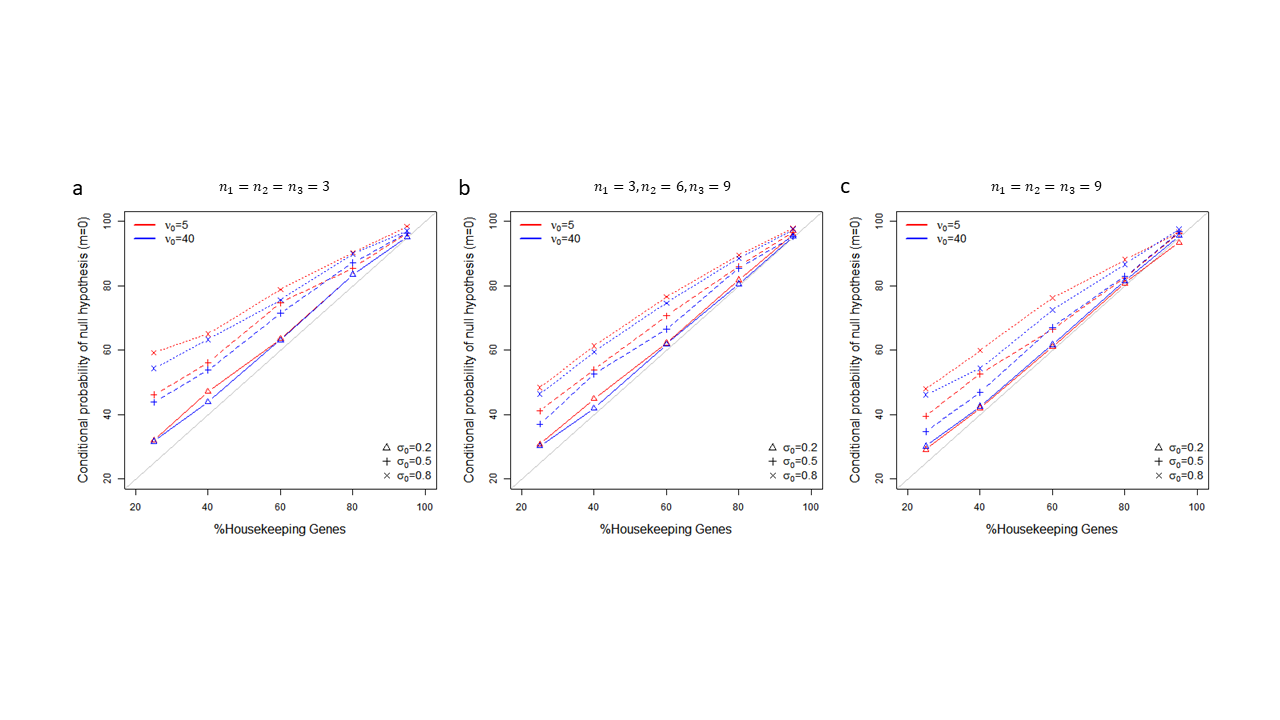
**

**Figure S3**. Comparisons of estimated conditional probabilities of the null hypothesis $H_{\text{SDEG, }0}^{null}$, $P\left\{ H_{\text{SDEG, }0}^{null} | H_{\text{SDEG}}^{null} \right\}$, versus the true proportions of housekeeping genes, under different settings of noisy scenarios and sample sizes.

**
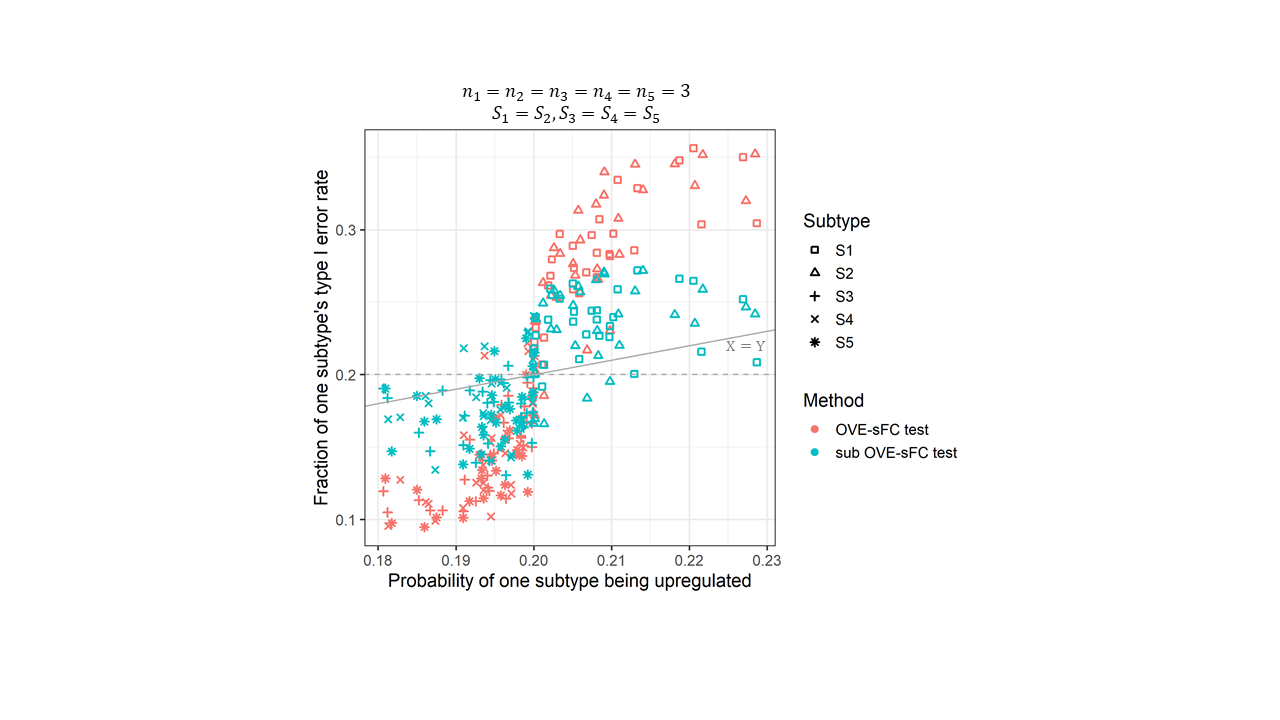
**

**Figure S4**. Fraction of type I error in each of five subtypes, versus probability of each subtype being upregulated under $H_{\text{SDEG}}^{null}$ (estimated by Eq. S9). Each point is associated with one of the simulation settings (housekeeping genes: 95%, 80%, 60%, 40%, or 25%; $\sigma_{0}$: 0.2, 0.5, or 0.8; $\nu_{0}$: 5, or 40). Sample size is three per subtype. The first two subtypes are drawn from the same one population and the remaining three drawn from another.

**
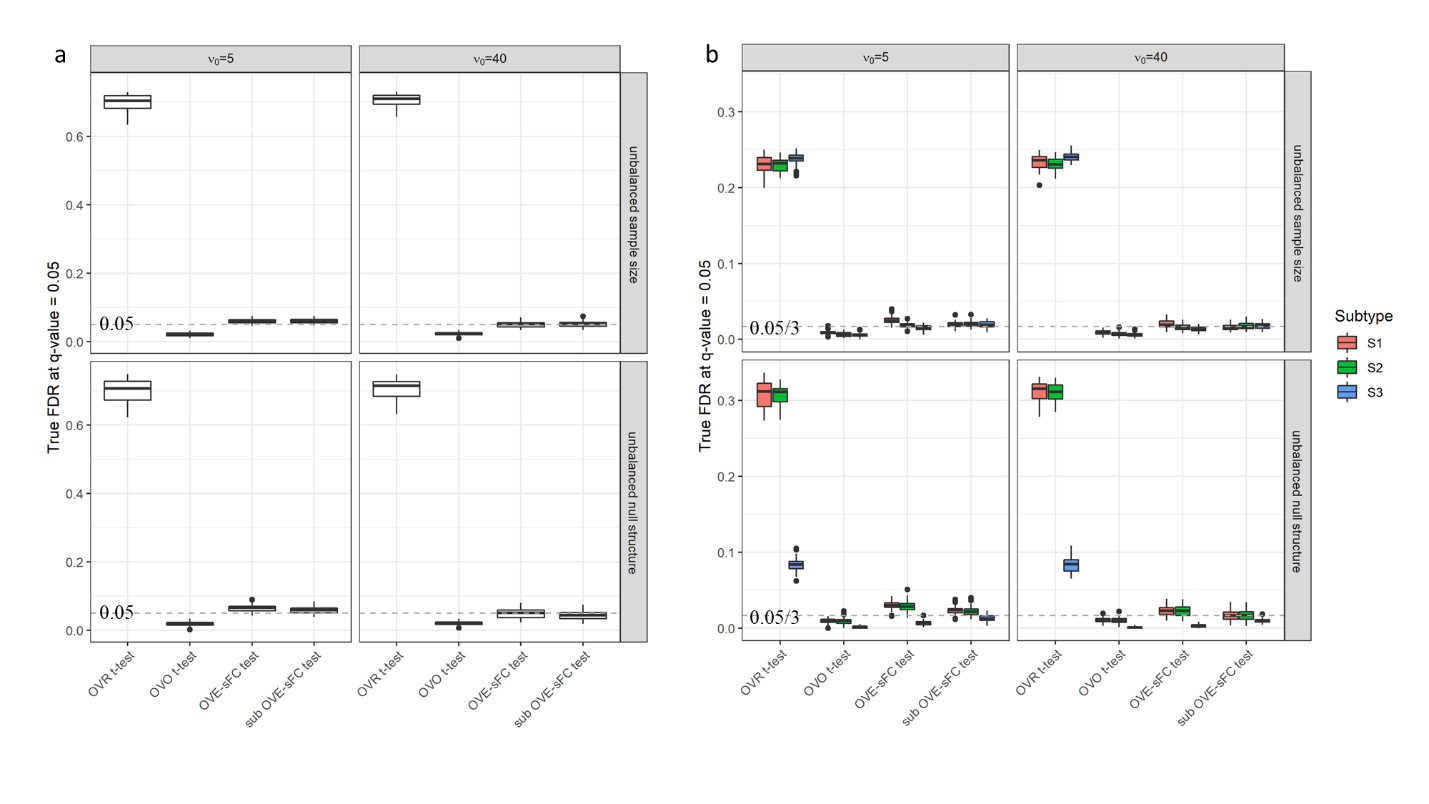
**

**Figure S5**. FDR control under the multiple simulation settings with three unbalanced subtypes. (a) True FDR at q-value =0.05 across all subtypes (dash line is at 0.05); (b) True FDR at q-value =0.05 in each subtype (dash line is at 0.05/3).

**
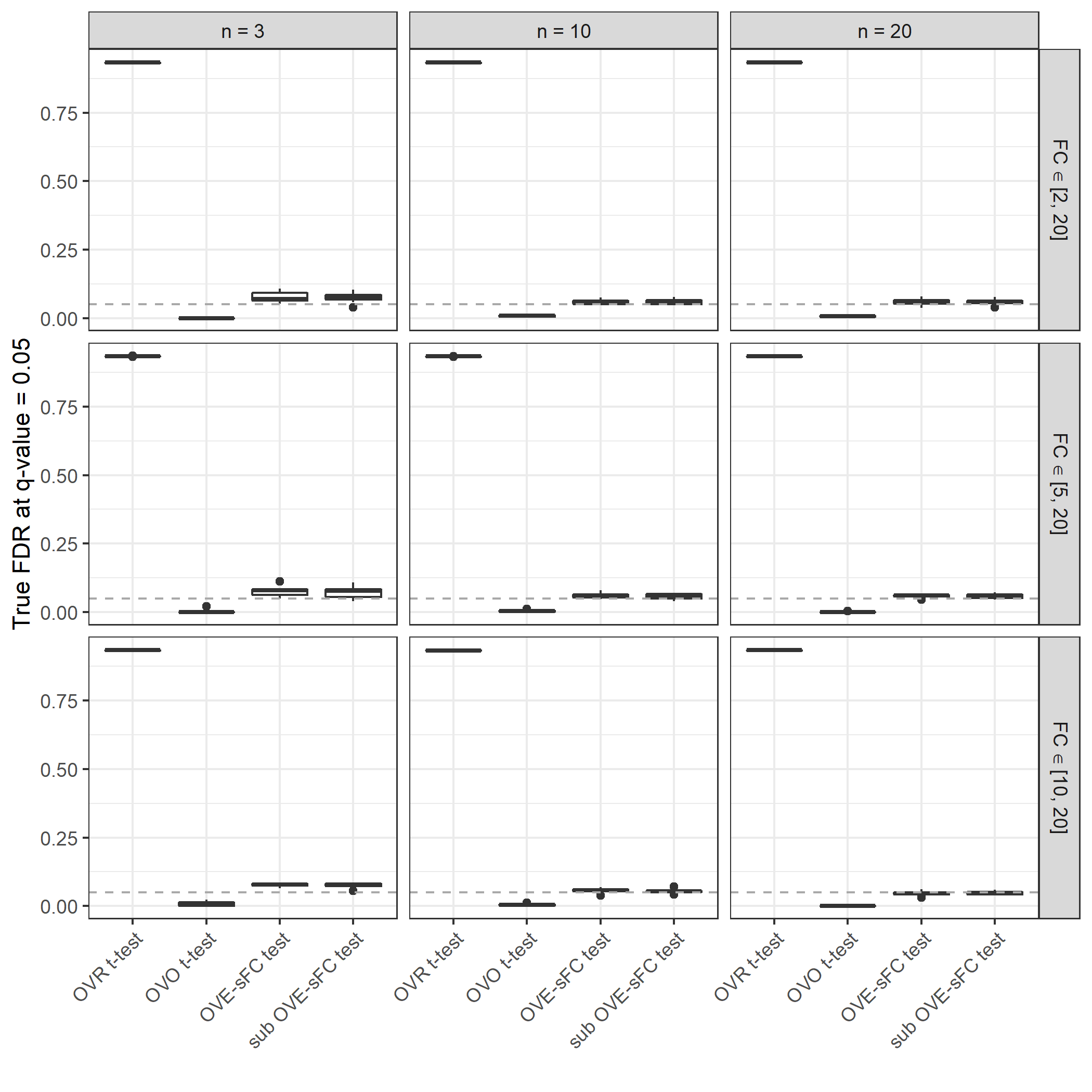
**

**Figure S6**. FDR control under the multiple simulation settings in RNAseq-derived simulations (dash line is at 0.05). Sample size is 3, 10 or 20 per subtype. Fold change range of SDEGs is [2,20], [5,20], or [10,20]. Non-SDEG distribution is consistent with the base real dataset under the null hypothesis. Each simulation setting is repeated 20 times


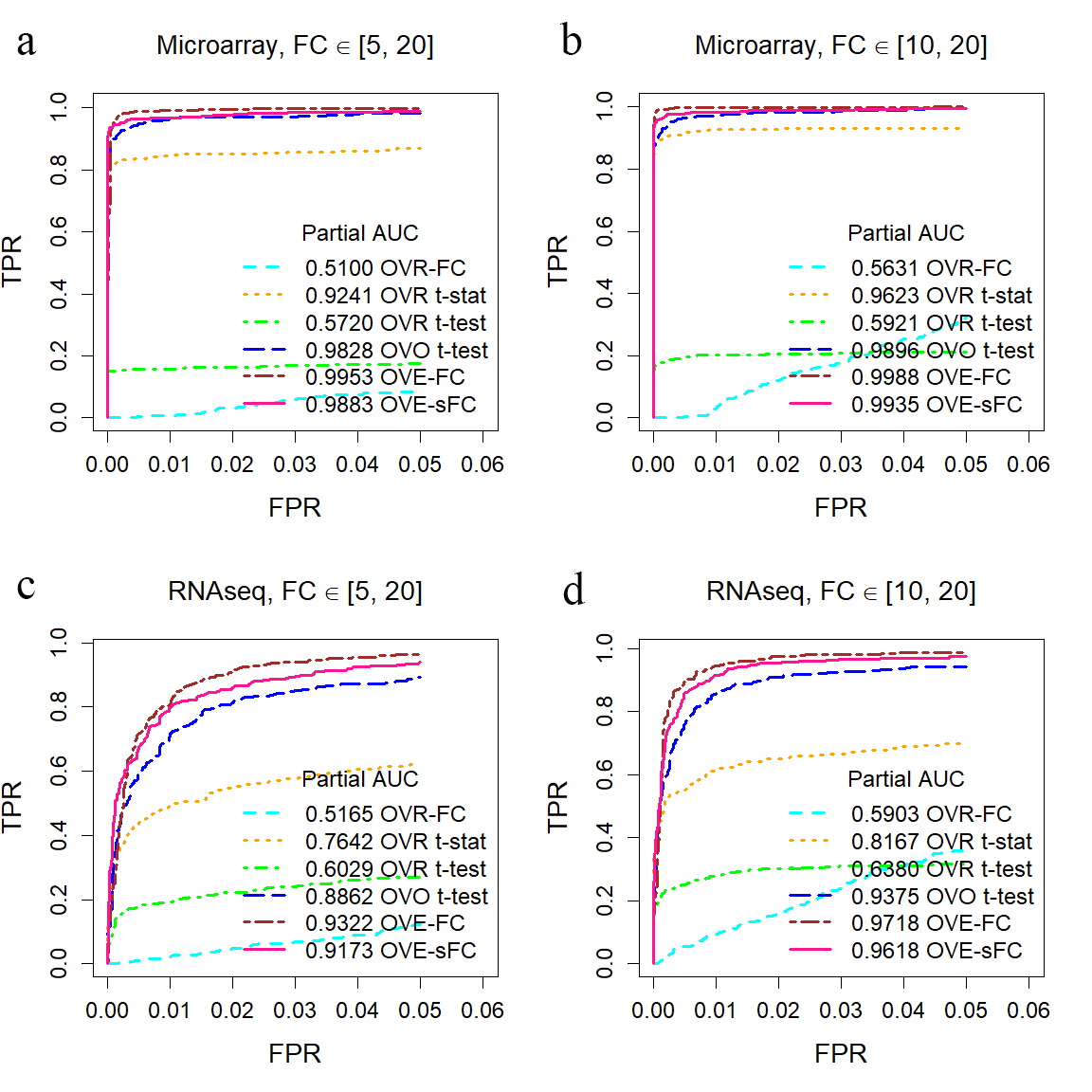


**Figure S7**. Assessment on detection power (partial ROC curves, FPR < 0.05) using real data derived simulations (data distribution is consistent with the base real dataset under the null hypothesis) involving seven unbalanced subtypes with various parameter settings. Sample size is 3 per subtype. (a)(b) partial ROC curves across different FPR points on microarray-derived data. (c)(d) partial ROC curves across different FPR points on RNAseq-derived data.

**
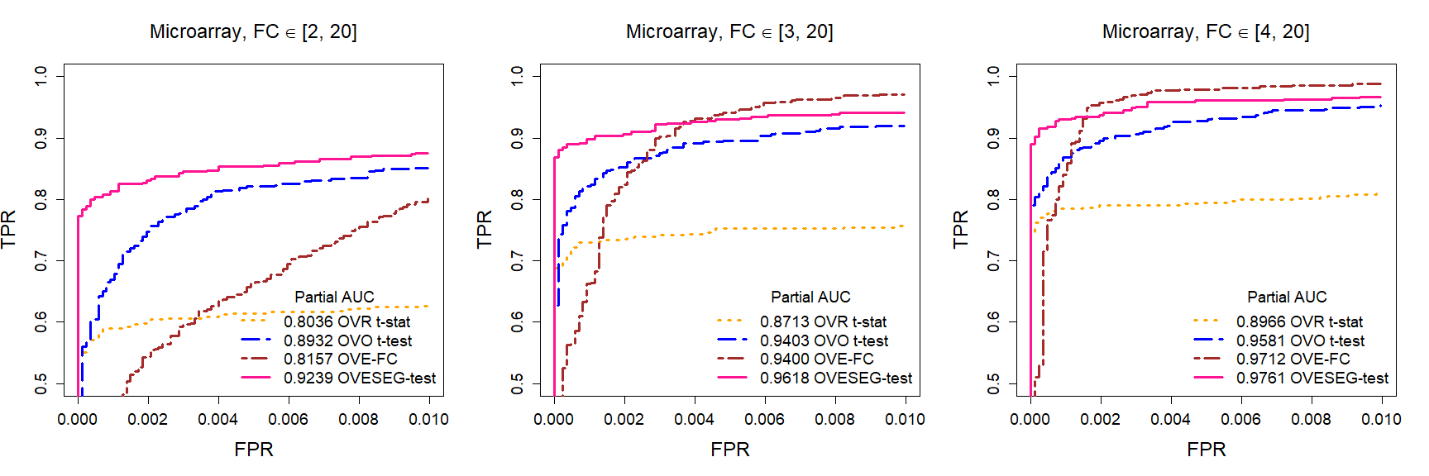
**

**Figure S8**. Comparative assessment on detection power (partial ROC curves, FPR < 0.01) using simulations produced from real gene expression data (data distribution is consistent with the baseline real gene expression dataset under the null hypothesis) involving seven imbalanced subtypes and less ideal SDEGs with smaller fold change. Sample size is 3 per subtype. The effects are made smaller to lower the TPR, with the lower end of SDEG fold-change starting 2, 3, 4. It can be seen that that OVE-sFC clearly outperforms OVO t-test in all scenarios with different effect size, and OVE-FC clearly outperforms all other peer methods except OVO t-test only when effect size is very small. It is worth mentioning that, because OVE-FC neither considers the variance term in the test nor borrows the relevant/useful information cross genes in estimating null distribution, OVE-FC expectedly underperforms OVO t-test when effect/sample size is small; and precisely for this reason, we developed OVE-sFC.

**
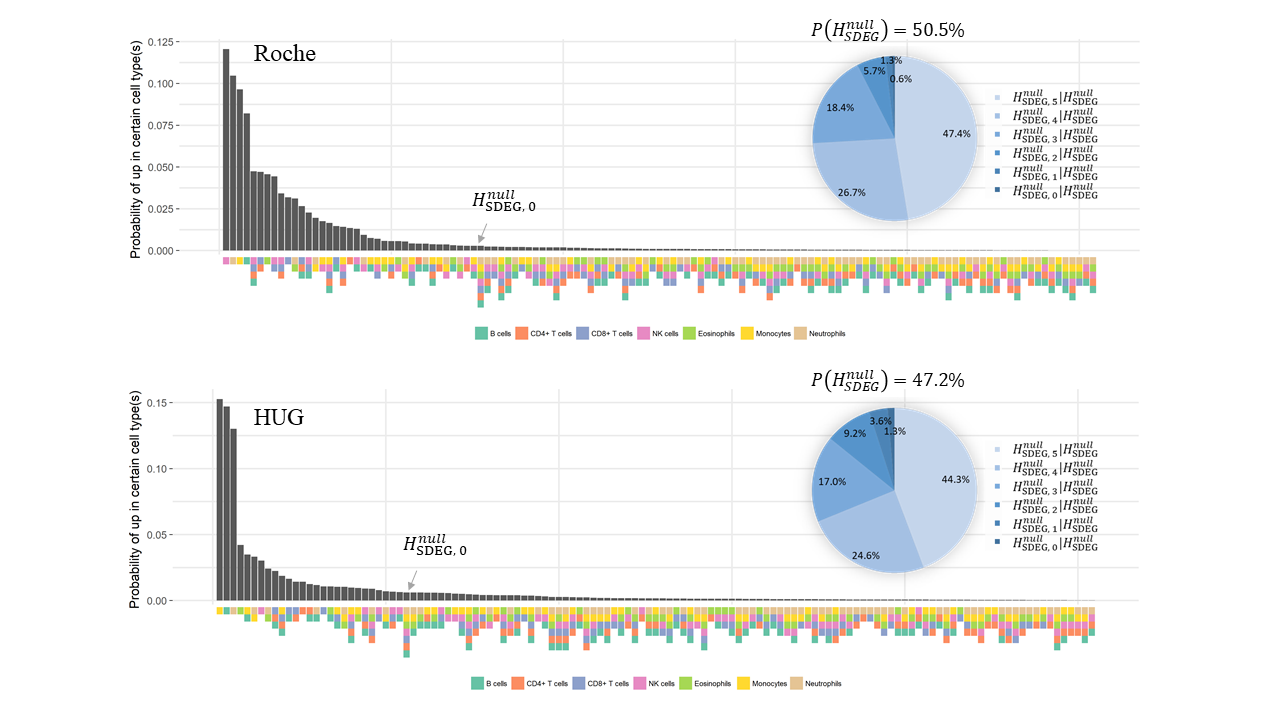
**

**Figure S9**. Landscape of 127 ($=2^{7}-1$) gene expression patterns. Estimated probabilities of exclusive expression in any certain cell type(s) are ordered in decreasing value. The pie charts show the conditional probability of each the null hypothesis.

**
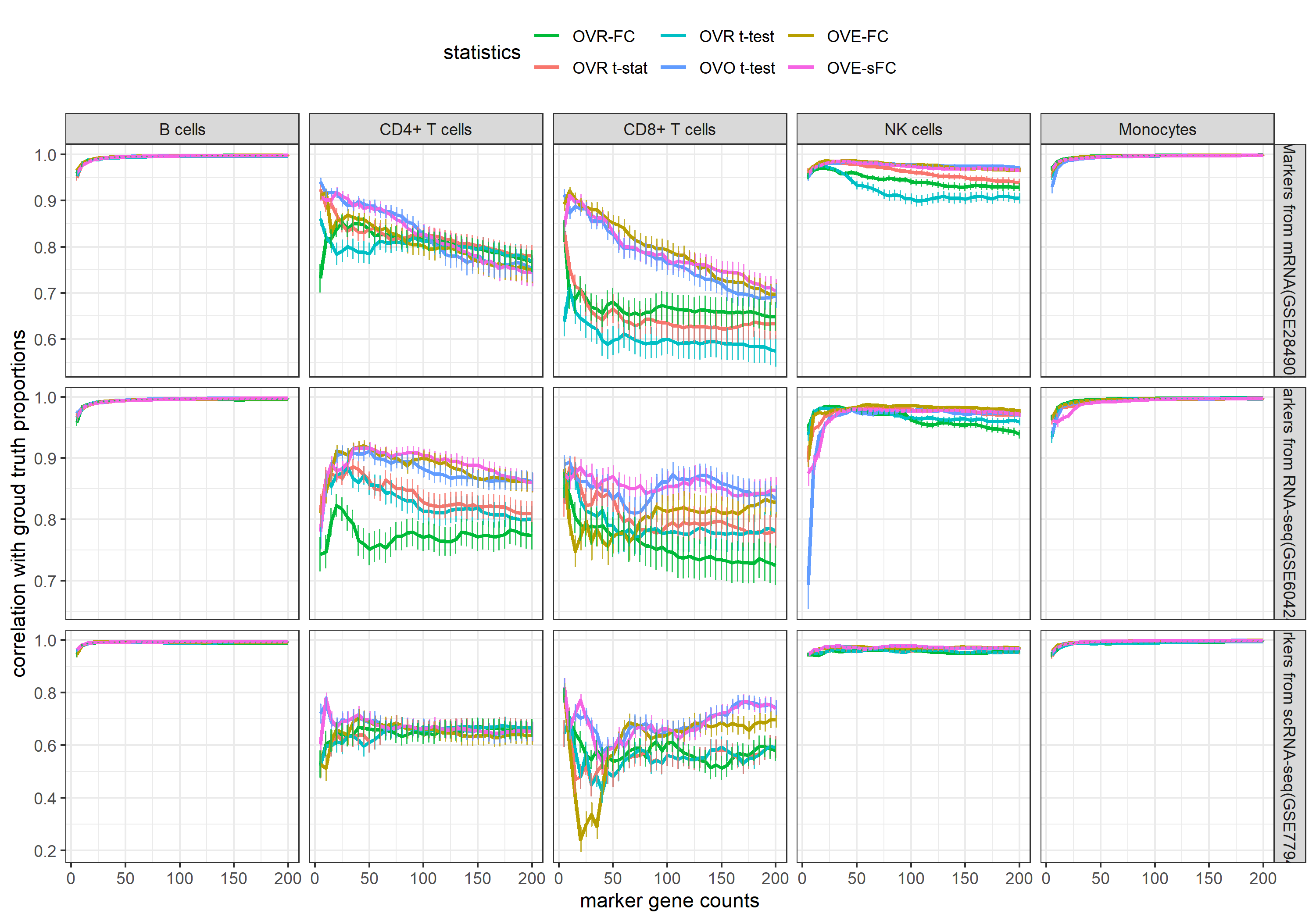
**

**Figure S10**. Correlation coefficients between CAM score and ground truth proportion for each cell type, with score estimated by a fixed number of markers from independent dataset to quantify subtypes in heterogeneous samples simulated by mixing purified mRNA expression levels in GSE28491. Mean and 95% confidence interval are computed among 20 repeated experiments.

**
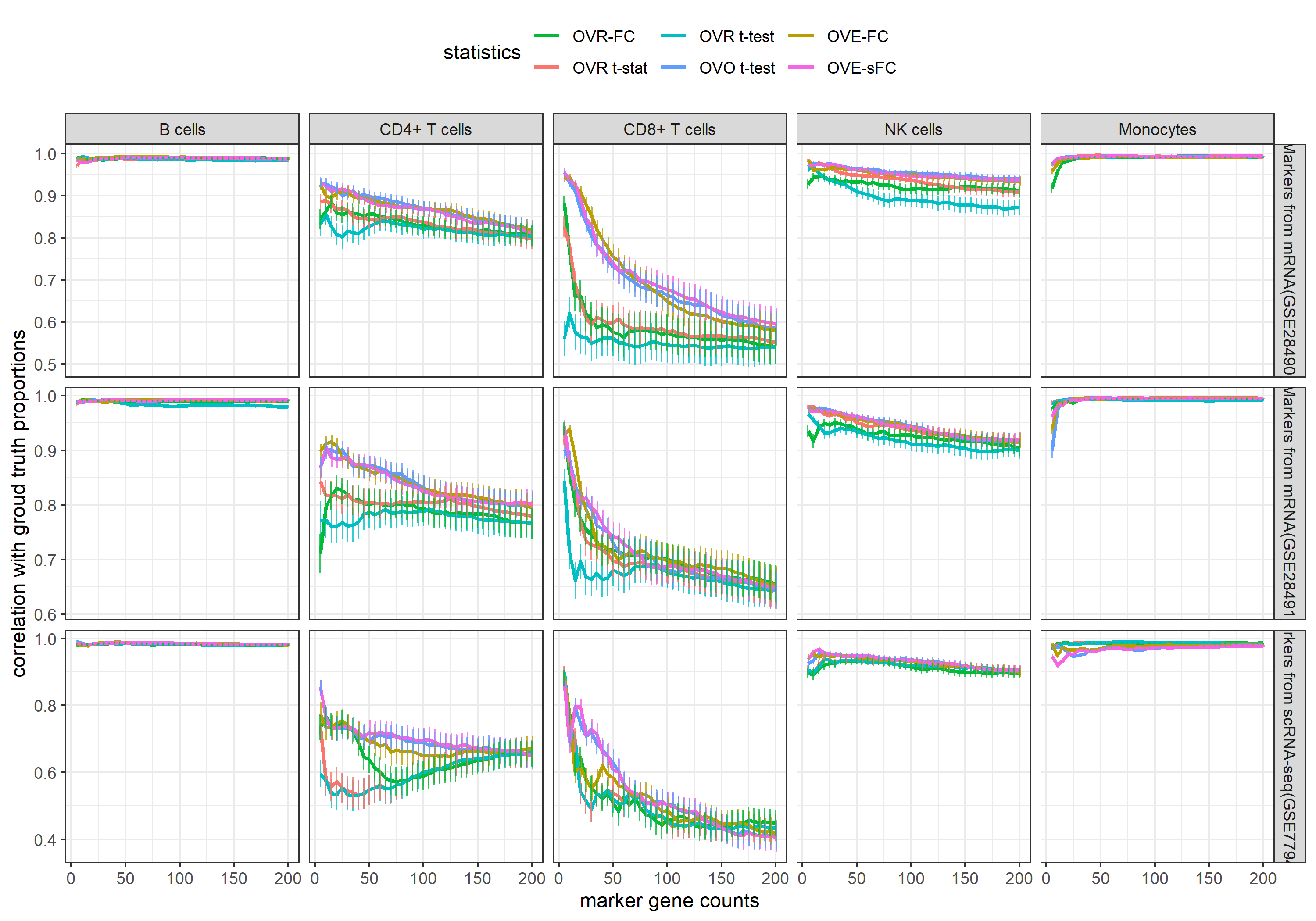
**

**Figure S11.** Correlation coefficients between CAM score and ground truth proportion for each cell type, with score estimated by a fixed number of markers from independent dataset to quantify subtypes in heterogeneous samples simulated by mixing purified RNAseq counts in GSE60424. Mean and 95% confidence interval are computed among 20 repeated experiments.

## References

1. Yu, G. et al. Matched Gene Selection and Committee Classifier for Molecular Classification of Heterogeneous Diseases. *J. Mach. Learn. Res.* **11**, 2141-2167 (2010).

2. Guo, X. & Pan, W. Using weighted permutation scores to detect differential gene expression with microarray data. *Journal of Bioinformatics and Computational Biology* **03**, 989-1006 (2005).

3. Strimmer, K. fdrtool: a versatile R package for estimating local and tail area-based false discovery rates. *Bioinformatics* **24**, 1461-1462 (2008).

4. Kulinskaya, E. On two-sided p-values for non-symmetric distributions. *arXiv preprint arXiv:0810.2124* (2008).

5. Zhou, Y.-H. & Wright, F.A. Hypothesis testing at the extremes: fast and robust association for high-throughput data. *Biostatistics* **16**, 611-625 (2015).

6. Smyth, G.K. Linear models and empirical bayes methods for assessing differential expression in microarray experiments. *Statistical applications in genetics and molecular biology* **3**, Article3 (2004).

7. Law, C.W., Chen, Y., Shi, W. & Smyth, G.K. voom: precision weights unlock linear model analysis tools for RNA-seq read counts. *Genome Biology* **15**, R29 (2014).

8. Suomi, T., Seyednasrollah, F., Jaakkola, M.K., Faux, T. & Elo, L.L. ROTS: An R package for reproducibility-optimized statistical testing. *PLoS computational biology* **13**, e1005562 (2017).

9. Wang, M., Master, S.R. & Chodosh, L.A. Computational expression deconvolution in a complex mammalian organ. *BMC Bioinformatics* **7**, 328-328 (2006).

10. Kuhn, A., Thu, D., Waldvogel, H.J., Faull, R.L. & Luthi-Carter, R. Population-specific expression analysis (PSEA) reveals molecular changes in diseased brain. *Nature methods* **8**, 945-947 (2011).

11. Allantaz, F. et al. Expression Profiling of Human Immune Cell Subsets Identifies miRNA-mRNA Regulatory Relationships Correlated with Cell Type Specific Expression. *PLoS ONE* **7**, e29979 (2012).

12. Finotello, F. & Trajanoski, Z. Quantifying tumor-infiltrating immune cells from transcriptomics data. *Cancer Immunology, Immunotherapy* **67**, 1031-1040 (2018).

13. Croux, C., Filzmoser, P. & Oliveira, M.R. Algorithms for Projection–Pursuit robust principal component analysis. *Chemometrics and Intelligent Laboratory Systems* **87**, 218-225 (2007).

14. Wang, N. et al. Mathematical modelling of transcriptional heterogeneity identifies novel markers and subpopulations in complex tissues. *Scientific reports* **6**, 18909 (2016).

15. Becht, E. et al. Estimating the population abundance of tissue-infiltrating immune and stromal cell populations using gene expression. *Genome Biology* **17**, 218 (2016).

16. Yoshihara, K. et al. Inferring tumour purity and stromal and immune cell admixture from expression data. *Nat Commun* **4** (2013).

17. Aran, D., Hu, Z. & Butte, A.J. xCell: digitally portraying the tissue cellular heterogeneity landscape. *Genome Biology* **18**, 220 (2017).

18. Schelker, M. et al. Estimation of immune cell content in tumour tissue using single-cell RNA-seq data. *Nat Commun* **8**, 2032 (2017).
